# 3D numerical simulation of acoustophoretic motion induced by boundary-driven acoustic streaming in standing surface acoustic wave microfluidics

**DOI:** 10.1038/s41598-021-90825-z

**Published:** 2021-06-25

**Authors:** Mohammad Sadegh Namnabat, Mahdi Moghimi Zand, Ehsan Houshfar

**Affiliations:** 1 Small Medical Devices, BioMEMS & LoC Lab, School of Mechanical Engineering, College of Engineering, University of Tehran, Tehran, Iran; 2 School of Mechanical Engineering, College of Engineering, University of Tehran, Tehran, Iran

**Keywords:** Engineering, Materials science, Mathematics and computing, Nanoscience and technology, Physics

## Abstract

Standing surface acoustic waves (SSAWs) have been widely utilized in microfluidic devices to manipulate various cells and micro/nano-objects. Despite widespread application, a time-/cost-efficient versatile 3D model that predicts particle behavior in such platforms is still lacking. Herein, a fully-coupled 3D numerical simulation of boundary-driven acoustic streaming in the acoustofluidic devices utilizing SSAWs has been conducted based on the limiting velocity finite element method. Through this efficient computational method, the underlying physical interplay from the electromechanical fields of the piezoelectric substrate to different acoustofluidic effects (acoustic radiation force and streaming-induced drag force), fluid–solid interactions, the 3D influence of novel on-chip configuration like tilted-angle SSAW (taSSAW) based devices, required boundary conditions, meshing technique, and demanding computational cost, are discussed. As an experimental validation, a taSSAW platform fabricated on YX 128 $^\circ$ LiNbO_3_ substrate for separating polystyrene beads is simulated, which demonstrates acceptable agreement with reported experimental observations. Subsequently, as an application of the presented 3D model, a novel sheathless taSSAW cell/particle separator is conceptualized and designed. The presented 3D fully-coupled model could be considered a powerful tool in further designing and optimizing SSAW microfluidics due to the more time-/cost-efficient performance than precedented 3D models, the capability to model complex on-chip configurations, and overcome shortcomings and limitations of 2D simulations.

## Introduction

Manipulation of microparticles is of utmost importance in a wide array of biomedical, biochemical, and biophysical applications^1,2,3^. Since the emergence of lab-on-a-chip (LoC) technologies, especially in the past two decades, different manipulation strategies, including electrokinetic, hydrodynamic, optical, magnetophoretic, and acoustophoretic based method, have been developed^4–9^. Each method has its portfolio of strengths and weaknesses, which makes them suitable for a particular application. However, acoustofluidic based microfluidic approaches have some definite advantages over competing technologies. Acoustofluidic methods are capable of cell/particle manipulation based on relative density and compressibility and are not restricted by the target particles' electric, magnetic, and optical properties. Moreover, excellent cell/microorganism viability, contact/label-free manipulation, relatively simple and compact devices, and versatility to readily integrate with other microfluidic technologies make acoustofluidic approaches, especially standing surface acoustic wave (SSAW) based methods, a promising platform for the future of LoC technologies, and, consequently, the focus of numerous experimental and theoretical investigations^10–12^. To fully grasp the potential of acoustofluidic devices, further development in the reliability and efficiency of the available devices is mandatory, which can be achieved through optimization and conceiving innovative device design. Here, the numerical simulation could play a decisive role not only by providing a profound insight into the underlying physical interplay of acoustofluidic processes but also through considerably facilitating the cumbersome experimental and iterative process of improving device design, including a series of time-consuming and costly creating, fabricating, and testing measures. However, the simulation of acoustofluidic devices considering their detailed intricacies is prohibitively expensive due to the multiscale characteristics of involved parameters, including magnitude, time, and length scale difference, besides coupled multi-physics intrinsic of the problem^13^.

Nyborg’s perturbation expansion for all field variables has been proposed to overcome multiscale issues about the magnitude and time scale-difference in the acoustofluidic problem^14,15^. The fluid's behavior is studied by dividing the solution into time-harmonic and time-averaged response and implementing the first-order and second-order expansion in the governing equations. Due to the high computational cost of the method, which mainly arises from the required extremely fine mesh to capture boundary layer streaming, most conducted simulations are confined to a 2D simplification in which only a cross-sectional area of the fluid chamber is considered. Nevertheless, the staple factors contributing to the deviation of simplified 2D models from real acoustofluidic devices could be listed as follows; first, incorporating a numeric model of the piezoelectric substrate in the form of analytic expression based on prior knowledge of piezoelectric response as an unloaded substrate; second, the assumption of the perfect distribution of acoustic wave along the axis of the channel, and required approximation in adopting appropriate decay coefficient; third, neglecting the influence of lateral and structural vibration mode; fourth, ignoring the impact of different acoustic impedance and speed; fifth, dismissing the influence of acoustic absorption at the channel outlets; sixth, neglecting the influence of microchannel geometry, fluid flow characteristics, and IDT's structure on device performance^5,13,16–18^. Additionally, the final performance of an acoustofluidic device in particle manipulation (separation, focusing) under a specific condition is evaluated based on the inter-particle distance at the channel outlet, which would not be determined unless a 3D simulation of the acoustofluidic device was conducted. By the way, due to the computational limitations of the numeric models developed based on the perturbation approach, the conducted 3D numerical simulations are restricted to microchannels with a simple structure, which would be advantageous in capturing complicated acoustic streaming pattern, like in-plane streaming-flow rolls or butterfly pattern, not the overall performance of an acoustofluidic device utilizing a complex on-chip configuration^13,19,20^. In the presented fully-coupled 3D model of SSAW microfluidics, all the aforementioned shortcomings of precedented simulations are overcome.

Resolving the boundary layer streaming is essential for deriving the acoustic streaming in the bulk of the microfluidic chamber since it provides the main driving force. To date, time-/cost-efficient simulation of boundary-driven acoustic streaming in 3D cases is only feasible by utilizing a semi-analytical approach known as the classic limiting velocity method^16,21,22^. Here, to the best of the author’s knowledge, for the first time, the limiting velocity finite element method is utilized to simulate SSAW microfluidics in a 3D manner. The developed 3D fully-coupled model would span every component of the device from the underlying physical interplay to electromechanical fields of the piezoelectric substrate, fluid–solid interactions, acoustic fields inside the microchannel and substrate, different acoustofluidic effects including the acoustophoretic motion of the particles resulting from acoustic radiation force and streaming-induced drag force, and the 3D influence of novel on-chip configurations. The necessity of such 3D fully-coupled modeling would become more conspicuous for design and optimization of complex on-chip configuration, like taSSAW microfluidics, where available 2D and 3D models could not capture the interplay impressions of complex geometrical parameters and multiscale involved physics on device performance. Therefore, to validate the presented simulation, a taSSAW microfluidic device investigated experimentally by Ding et al.^23^ is opted here, and it is demonstrated that the results are consistent with experimental observations. In the presented comprehensive numerical simulation, the influence of key variables including flow rate, operational frequency, input power (driving voltage), the tilt angle of the pressure nodal lines, particle, fluid, substrate, and channel wall properties on the device performance is examined. Afterward, as an application of the model to further improve the design and optimization of acoustofluidic devices, an on-chip configuration is proposed and evaluated to substitute sheath flows with SSAW in the prerequisite concentration step of the particle separation process in taSSAW based devices. This paper demonstrates how an appropriate 3D model of SSAW-driven devices based on the robust scientific basis would provide a general framework to systematically further the development, designing, and optimization of this type of acoustofluidic systems, which cannot be achieved solely by experiments due to the highly complex interplay among multiscale involved physics and existence of various geometrical, rheological, and physical properties.

### SSAW acoustofluidic system and governing equations

**Figure 1:**
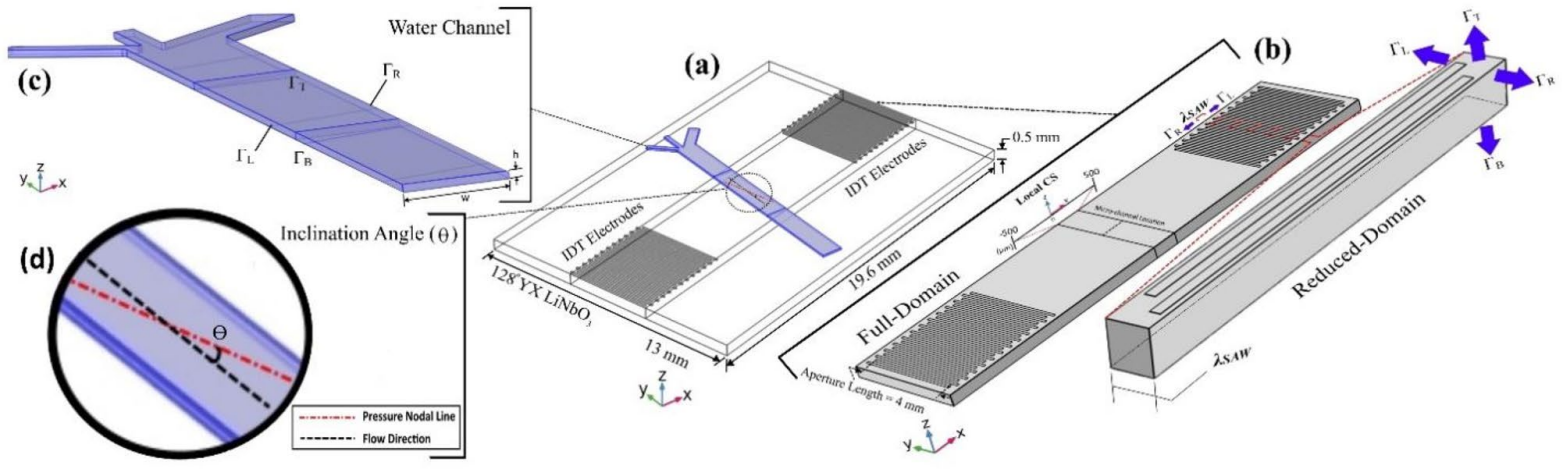
**(a)** Isometric view from the full 3D model of taSSAW acoustofluidic device, **(b)** full-domain and reduced-domain 3D model of the substrate, the computational domain boundaries, and considered local CS for substrate polarization, **(c)** fluid cavity boundaries including ${\Gamma }_{L}$, ${\Gamma }_{R}$, ${\Gamma }_{T}$ in contact with PDMS wall, and ${\Gamma }_{B}$ vibrating boundary in contact with LiNbO_3_ substrate, **(d)** inclination angle ($\theta$).

Figure 1 demonstrates the on-chip configuration and computational domain of a ta-SSAW acoustofluidic device with a hydrodynamic concentration approach. In line with Ding et al.^23^, a simple IDT structure with 24 pairs of electrodes, an aperture length (working region) of 4 mm, and an IDT pitch ($P$) of 400 μm (SAW wavelength (${\lambda }_{SAW}$) = $P$) fabricated on a Lithium Niobate (LiNbO_3_) piezoelectric substrate with 128^o^ YX crystallographic cut is considered for the presented simulation. The width and height of the PDMS microchannel are $w$=1000 μm and $h$=75 μm, respectively. The alternating electric voltage is applied at a $\pi$ phase difference to the IDTs so that the counter-propagating SAWs would have constructive superposition and generate SSAW with the maximum displacement amplitude. The field equations governing the piezoelectric, fluid, and particle behavior in SSAW microfluidics are briefly presented in the following.

### SAW propagation in piezoelectric solid

The wave equation for the mechanical displacement $\mathbf{u}=\mathbf{u}\left({\varvec{x}},t\right)$ of an elastic, homogeneous, isotropic as well as anisotropic medium without considering body forces is expressed as,

1 $$ \rho \frac{{\partial^{2} {\mathbf{u}}}}{{\partial t^{2} }} - \nabla .{\mathbf{T}} = 0 $$

where $\rho$ and ${\varvec{T}}=({T}_{ij})$ stand for material density and stress tensor, respectively. The linear constitutive equation of piezoelectric material in the stress-charge form is as follows.

2 $$ T_{ij} ({\mathbf{u}},{\mathbf{E}}) = c_{ijkl} S_{kl} ({\mathbf{u}}) - e_{kij} E_{k} $$

3 $$ D_{i} ({\mathbf{u}},{\mathbf{E}}) = e_{ikl} S_{kl} ({\mathbf{u}}) + \varepsilon_{ij} E_{j} $$

Here, ${\varvec{S}}=\left({S}_{kl}\right)=\boldsymbol{ }\left(\nabla {\varvec{u}}+{(\nabla {\varvec{u}})}^{T}\right)/2$ is linearized strain tensor, ${\varvec{c}}=({c}_{ijkl})$, ${\varvec{e}}=({e}_{ikl})$, and ${\varvec{\varepsilon}}=({\varepsilon }_{ij})$ refer to the positive definite fourth-order elasticity tensor, third-order stress piezoelectric tensor, and permittivity tensor, respectively. The electrical field (${\varvec{E}}=({E}_{k})$) and electrical displacement field (${\varvec{D}}=({D}_{i})$) are coupled with the mechanical field through Eqs. (2) and (3). By implementing a well-known quasi-static assumption in piezoelectric materials, Maxwell’s second equation reduces to $\nabla \times {\varvec{E}}=0$. Consequently, the electric field is irrotational and can be stated as the gradient of a scalar electric potential by ${\varvec{E}}= -\nabla \phi$. Furthermore, piezoelectric substrates can be considered perfect insulators, which means that the density of free electric charges on their surface can be neglected^24^. Therefore, the only relevant Maxwell’s equation is expressed as,

4 $$ \nabla .{\mathbf{D}} = 0 $$

By inserting Eq. (2) in Eq. (1) and Eq. (3) in Eq. (4), the linear field coupled equation for propagation of SAW in a piezoelectric substrate is obtained as,

5 $$ \rho \ddot{u}_{i} - c_{ijkl} u_{k,lj} - e_{kij} \phi_{,kj} = 0 $$

6 $$ e_{ikl} u_{k,li} - \varepsilon_{ij} \phi_{,ji} = 0 $$

### The first- and second-order fields of acoustically driven fluids

The well-known Navier–Stokes equations govern the motion of a linear viscous compressible fluid without considering body forces are given by,

7 $$ \rho_{f} (\dot{v}_{i} + v_{j} v_{i,j} ) = - p_{,i} + (4/3\eta + \eta_{B} )v_{j,ji} + \eta (v_{i,jj} - v_{j,ji} ) $$

where $p$, ${v}_{i}$, $\eta$, and ${\eta }_{B}$ stand for pressure and velocity field, dynamic, and bulk viscosity, respectively. By applying the perturbation approach on Eq. (7) and considering a harmonic time dependence for the acoustic field, the first-order time-harmonic equation of fluid motion, known as the lossy Helmholtz equation for the first-order pressure field ${p}_{1}$, is derived as^15^,

8 $$ p_{1,ii} = - k^{2} p_{1} $$

where $k=\left(1+i\gamma \right){k}_{0}=\left(1+i\gamma \right)\omega /{c}_{0}$ is the complex-valued wave number, and $\gamma$ and ${c}_{0}$ are the viscous damping factor and real-valued speed of sound in the fluid, respectively. Helmholtz equation for a damped wave is expressed by Eq. (8); however, as $\gamma \ll 1$ for water at 25 °C and MHz frequency range^15^, the viscous damping for acoustic wave propagation in the bulk of the fluid is neglected here. By solving Eq. (8) on the computational domain for ${p}_{1}$, the first-order velocity field ${{\varvec{v}}}_{1}$ is then calculated based on Eq. (9) in which ${\rho }_{0}$ is the quiescent fluid density.

9 $$ v_{1i} = - i\frac{1}{{\rho_{0} \omega }}p_{1,i} $$

Neglecting the inertia terms due to the typically low Reynolds numbers of the fluid flow in the microfluidic devices besides considering incompressible fluid, the governing equations for the mass and momentum balance laws in which the streaming second-order velocity field and pressure field are denoted as ${v}_{2}$ and ${p}_{2}$ are as follows.

10 $$ p_{2,i} = \eta v_{2i,jj} $$

11 $$ v_{2i,j} = 0 $$

### Acoustophoresis of suspended particles

The acoustophoretic motion in an established acoustic field is governed predominately by the acoustic radiation force (ARF) and Stokes drag force (DF) applied simultaneously by the first- and second-order fields, respectively. Herein, Settnes and Bruus's^25^ formulation of the ARF on a compressible and spherical particle suspended in a viscous fluid, developed based on the acoustic field scattering due to particles' presence, is adopted. By neglecting mutual interactions between particles, the ARF (${{\varvec{F}}}^{rad}$) on a spherical particle with radius $r$ ($r\ll {\lambda }_{SAW}$), density ${\rho }_{p}$, and compressibility ${\kappa }_{p}$ in a viscous fluid is expressed as^25^,

12 $$ {\varvec{F}}^{rad} = - \pi r^{3} \left[ {\frac{{2\kappa_{f} }}{3}{\text{Re}} \left[ {f_{1}^{*} p_{1}^{*} \nabla p_{1} } \right] - \rho_{f} {\text{Re}} \left[ {f_{2}^{*} {\varvec{v}}_{{\mathbf{1}}}^{*} .\nabla {\varvec{v}}_{{\mathbf{1}}} } \right]} \right] $$

where ${\kappa }_{f}$ is the compressibility of the fluid, and the dimensionless scattering coefficients ${f}_{1}$ and ${f}_{2}$ take the form of,

13a $$ f_{1} \left( {\tilde{\kappa }} \right) = 1 - \tilde{\kappa }{\text{ with }}\tilde{\kappa } = \frac{{\kappa_{p} }}{{\kappa_{f} }} $$

13b $$ f_{2} \left( {\tilde{\rho },\tilde{\delta }_{\upsilon } } \right) = \frac{{2\left[ {1 - \Lambda \left( {\tilde{\delta }_{\upsilon } } \right)} \right]\left( {\tilde{\rho } - 1} \right)}}{{2\tilde{\rho } + 1 - 3\Lambda \left( {\tilde{\delta }_{\upsilon } } \right)}}{\text{ with }}\tilde{\rho } = \frac{{\rho_{p} }}{{\rho_{f} }} $$

13c $$ \Lambda \left( {\tilde{\delta }_{\upsilon } } \right) = - \frac{3}{2}\left[ {1 + i\left( {1 + \tilde{\delta }_{\upsilon } } \right)} \right]\tilde{\delta }_{\upsilon } {\text{ with }}\tilde{\delta }_{\upsilon } = \frac{{\delta_{\upsilon } }}{r} $$

COMSOL Multiphysics does not contain a specific node to calculate the ARF, according to Eqs. (12) and (13); therefore, the required calculations are conducted with the developed code based on COMSOL’s internal functions and variables explained in Appendix B as Electronic Supplementary Information (ESI).

Under the assumption of negligible wall effect, Stokes DF (${{\varvec{F}}}^{drag}$) on a spherical particle with radius $r$ and velocity of ${{\varvec{v}}}^{{\varvec{p}}}=({v}_{i}^{p})$ moving in a fluid with streaming velocity ${{\varvec{v}}}_{2}$ is given by,

14 $$ {\varvec{F}}^{drag} = 6\pi \eta r\left( {{\varvec{v}}_{{\mathbf{2}}} - {\varvec{v}}^{{\varvec{P}}} } \right) $$

By neglecting the inertial and buoyant forces, the motion of $jth$ particle with mass ${m}_{j}$ according to Newton’s second law is governed by,

$$ m_{j} \dot{v}_{j}^{p} = {\varvec{F}}^{{{\varvec{drag}}}} + {\varvec{F}}^{rad} $$

## Numerical simulation

The field equations and the associated boundary conditions in the simulated acoustofluidic systems are numerically solved utilizing the finite-element (FE) package COMSOL Multiphysics 5.3a^26^. The values of implemented physical properties for the piezoelectric substrate (YX 128° LiNbO3), fluid medium (water), and suspended particles/cells (polystyrene beads, WBCs, MCF-7 breast cancer cells) are listed in Table 1. The elasticity, piezoelectric, and permittivity tensor constants of LiNbO3 with 128° YX crystallographic cut are calculated based on the necessary matrix operations, explained by Auld^27^, with developed MATLAB code. Appendix A in ESI is dedicated to a detailed description of the procedure. This section is devoted to the explanation of essential details concerning the conducted simulation.

**Table 1:** Material parameters (at T = 25 °C).

**Polystyrene**			
Density^28^ Poisson’s ratio^29^ Speed of sound (at 20 °C)^30^ Compressibility^a^	${\rho }_{ps}$ ${\sigma }_{ps}$ ${c}_{ps}$ ${\kappa }_{ps}$	1050 0.35 2350 1.72 $\times$ 10^–10^	kg m^−3^ – m s^−1^ Pa^−1^
**Poly-dimethyl siloxane (PDMS, 10: 1)**			
Density^31^ Speed of sound^32^ Attenuation coefficient (9 MHz)^32^	${\rho }_{wall}$ ${c}_{wall}$ ${\alpha }_{s,wall}$	920 1076.5 47.85	kg m^−3^ m s^−1^ dB cm^−1^
**Lithium niobate (YX 128° LiNbO**_**3**_**)**			
Density^23^ Speed of sound^23^	${\rho }_{sub}$ ${c}_{sub}$	4650 3997	kg m^−3^ m s^−1^
**Water**			
Density^26^ Speed of sound^26^ Dynamic viscosity^26^ Bulk viscosity^33^ Compressibility^b^	${\rho }_{f}$ ${c}_{f}$ $\eta$ ${\eta }_{B}$ ${\kappa }_{f}$	997 1497 0.890 2.47 4.48 $\times$ 10^–10^	kg m^−3^ m s^−1^ mPa s mPa s Pa^−1^
**Leukocytes (WBCs) **^23^			
Density Compressibility Mean diameter	${\rho }_{WBC}$ ${\kappa }_{WBC}$ ${d}_{WBC}$	1019 3.99 $\times$ 10^–10^ 12	kg m^−3^ Pa^−1^ μm
**Breast cancer cells (MCF-7) **^23^			
Density Compressibility Mean diameter	${\rho }_{MCF-7}$ ${\kappa }_{MCF-7}$ ${d}_{MCF-7}$	1068 4.22 $\times$ 10^–10^ 20	kg m^−3^ Pa^−1^ μm

^a^Calculated from Landau and Lifshitz as ${\kappa }_{ps}= \frac{3(1-{\sigma }_{ps})}{(1+{\sigma }_{ps})}\frac{1}{({\rho }_{ps}{c}_{ps}^{2})}$^34^.

^b^Calculated as $${\kappa }_{0}=\frac{1}{({\rho }_{0}{c}_{0}^{2})}$$.

### Modal and harmonic analysis of piezoelectric substrate

The simulation of SAW propagation in the piezoelectric substrate would be challenging due to the exhibited anisotropy of the material and intrinsic electromechanical coupling. Another demanding aspect of analyzing SAW propagation in a piezoelectric solid is the possibility of inducing various wave types (Rayleigh SAW or pseudo-SAW^35,36^) from one form of excitation^5,37^. Therefore, a two-step FE analysis including modal (eigenvalue based) analysis in the reduced-domain and frequency analysis in the full domain (Fig. 1b) is developed to ensure Rayleigh SAW propagated predominantly by emphasizing particle manipulation in acoustofluidic devices.

In the reduced-domain modal analysis, based on the periodic structure of fabricated IDTs on the substrate surface, the simulation is confined to a single wavelength of propagated SAW as the structure period (Fig. 1b). Utilizing modal analysis would help circumvent two main challenges, including decreasing computational cost (time and capacity) and deriving discernible vibration mode shapes. Due to considering a section of the computational domain in the modal analysis, the periodic boundary condition is utilized on the left and right boundaries (${\Gamma }_{L}$, ${\Gamma }_{R}$) of the computational domain (Fig. 1b) based on Eq. (16) to preserve the continuity of the analysis and validity of the results^38^ in which ${u}_{L}$, ${u}_{R}$, ${\phi }_{L}$, ${\phi }_{R}$ are the displacements and electric potentials on the left and right boundaries of the reduced-domain.

16a $$ u_{L} = u_{R} $$

16b $$ \phi_{L} = \phi_{R} $$

The stress-free boundary condition is applied to the top boundary (${\Gamma }_{T}$). The amplitude of Rayleigh waves decays exponentially with the substrate depth. Therefore, at the bottom of the domain (${\Gamma }_{B}$), the mechanical displacement (${\varvec{u}}$) and electric potential ($\phi$) would vanish as $z\to \infty$, which in turn prevents a reflection of the wave back to the domain. The harmonic analysis aims to specify operating frequency with the best pressure lines for particle manipulation by examining stress, displacement, and electric potential field in the micro-channel location on the substrate (Fig. 1b). Neglecting the periodic boundary condition, the mechanical boundary condition in the harmonic analysis is analogous to modal analysis. Considerations regarding mesh element size and type (Fig. S3) are brought in ESI as Appendix C.

### Limiting velocity method

Based on the well-established Rayleigh-Schlichting boundary layer theory for acoustic streaming^39^, the interaction between fluid medium and vibrating solid walls leads to the generation of a thin viscous boundary layer of thickness ${\delta }_{\upsilon }={\left(2\upsilon /\omega \right)}^{1/2}$ where $\omega$ and $\upsilon$ are angular frequency of SAW and kinematic viscosity, respectively. Capturing the boundary layer in a 3D numerical model is prohibitively expensive due to the domain's required fine discretization. In addressing this challenge, the streaming velocity distribution based on an analytical solution of the near boundary streaming, which is presented by Nyborg^40^ and with later modification by Lee and Wang^41^, just outside the boundary layer vortices can be utilized as a slip velocity boundary condition on ${\Gamma }_{B}$ boundary of the water channel in Fig. 1c, known as *limiting velocity,* for resolving the outer steady streaming flow field (Eqs. 10 and 11).

The limiting velocity on a planar fluid–solid interface normal to the z-axis is calculated based on first-order velocity field components traditionally denoted as ${u}_{a0}, {v}_{a0}$, and ${w}_{a0}$ in the literature. The notation is kept here to emphasize that the expression is calculated in the interface coordinate system, not a global one, where the tangential directions are $x$ and $y$, and the normal direction is $z$ corresponding to the velocity components ${u}_{a0}, {v}_{a0}$, and ${w}_{a0}$, respectively. The limiting velocity expression can be stated as^40,41^,

17a $$ \begin{gathered} u_{L} = - \frac{1}{4\omega }{\text{Re}} \left\{ {u_{a0} \frac{{du_{a0}^{*} }}{dx} + v_{a0} \frac{{du_{a0}^{*} }}{dy}} \right. \hfill \\ \, \left. { + u_{a0}^{*} \left[ {(2 + i)\left( {\frac{{du_{a0} }}{dx} + \frac{{dv_{a0} }}{dy} + \frac{{dw_{a0} }}{dz}} \right) - (2 + 3i)\frac{{dw_{a0} }}{dz}} \right]} \right\} \hfill \\ \end{gathered} $$

17b $$ \begin{gathered} v_{L} = - \frac{1}{4\omega }{\text{Re}} \left\{ {u_{a0} \frac{{dv_{a0}^{*} }}{dx} + v_{a0} \frac{{dv_{a0}^{*} }}{dy}} \right. \hfill \\ \, \left. { + v_{a0}^{*} \left[ {(2 + i)\left( {\frac{{du_{a0} }}{dx} + \frac{{dv_{a0} }}{dy} + \frac{{dw_{a0} }}{dz}} \right) - (2 + 3i)\frac{{dw_{a0} }}{dz}} \right]} \right\} \hfill \\ \end{gathered} $$

where the asterisk superscript, ^*^, denotes the complex conjugate value of the acoustic velocities. Due to considering fluid acoustic velocity without wall vibration in the interface tangential directions for the derivation of limiting velocity expressions, the difference between first-order fluid velocity outside the boundary layer (${v}_{x}$ and ${v}_{y}$ in Eq. (9)) and the vibration velocities of the substrate (${u}_{x}$ and ${u}_{y}$ are substrate displacement components in Eqs. (5) and (6)) is regarded as the interface tangential velocities ${u}_{a0}$ and ${v}_{a0}$ which can be expressed as^42^,

18a $$ u_{a0} = v_{1x} - \dot{u}_{x} $$

18b $$ v_{a0} = v_{1y} - \dot{u}_{y} $$

Nevertheless, due to the preserving continuity in the interface normal direction, no modification is required, and the velocity component ${w}_{a0}$ is identical to the fluid velocity component ${v}_{z}$ and substrate velocity component ${\dot{u}}_{z}$ in the normal direction, which can be written as^42^,

19 $$ w_{a0} = v_{1z} = \dot{u}_{z} $$

The calculation of limiting velocity based on Eq. (17) and corresponding required matrix operations utilizing COMSOL Multiphysics 5.3a internal functions and variables in a fluid–solid vibrating planar interface with arbitrary orientation is presented in Appendix B as ESI.

### Boundary conditions and computational cost

To observe conciseness, subtle considerations regarding acoustic boundary conditions and fluid–solid interactions for solving Helmholtz equation (Eq. 8) on the computational domain (Fig. 1a) besides analyzing computational demands which attest the time-/cost-efficiency of the presented 3D fully-coupled model of SSAW microfluidics compared to other available counterparts are presented in Appendix C as ESI.

## Results and discussion

### Optimal actuation frequency of Rayleigh SAW

Considering ${\lambda }_{SAW}$=400 μm and ${c}_{sub}$=3997 m/s (Table 1), the operating frequency would be in the 9 to 10 MHz frequency range. Therefore, eighty vibration mode shapes around 9.5 MHz frequency are investigated in the modal analysis, among which in twenty-two vibration mode shapes, Rayleigh SAW propagates in the substrate. Two main characteristics of Rayleigh waves are considered here to opt for appropriate mode shapes. First, most of the wave energy (90%) is confined within one wavelength from the substrate surface. Second, the substrate displacement profile would follow an elliptical pattern in which most of the polarization is confined to the sagittal plane ((x,z)-plane in Fig. 1b), and limited polarization in the transverse or frontal plane is tolerated (Fig. S4 in ESI)^24^.

**Figure 2:**
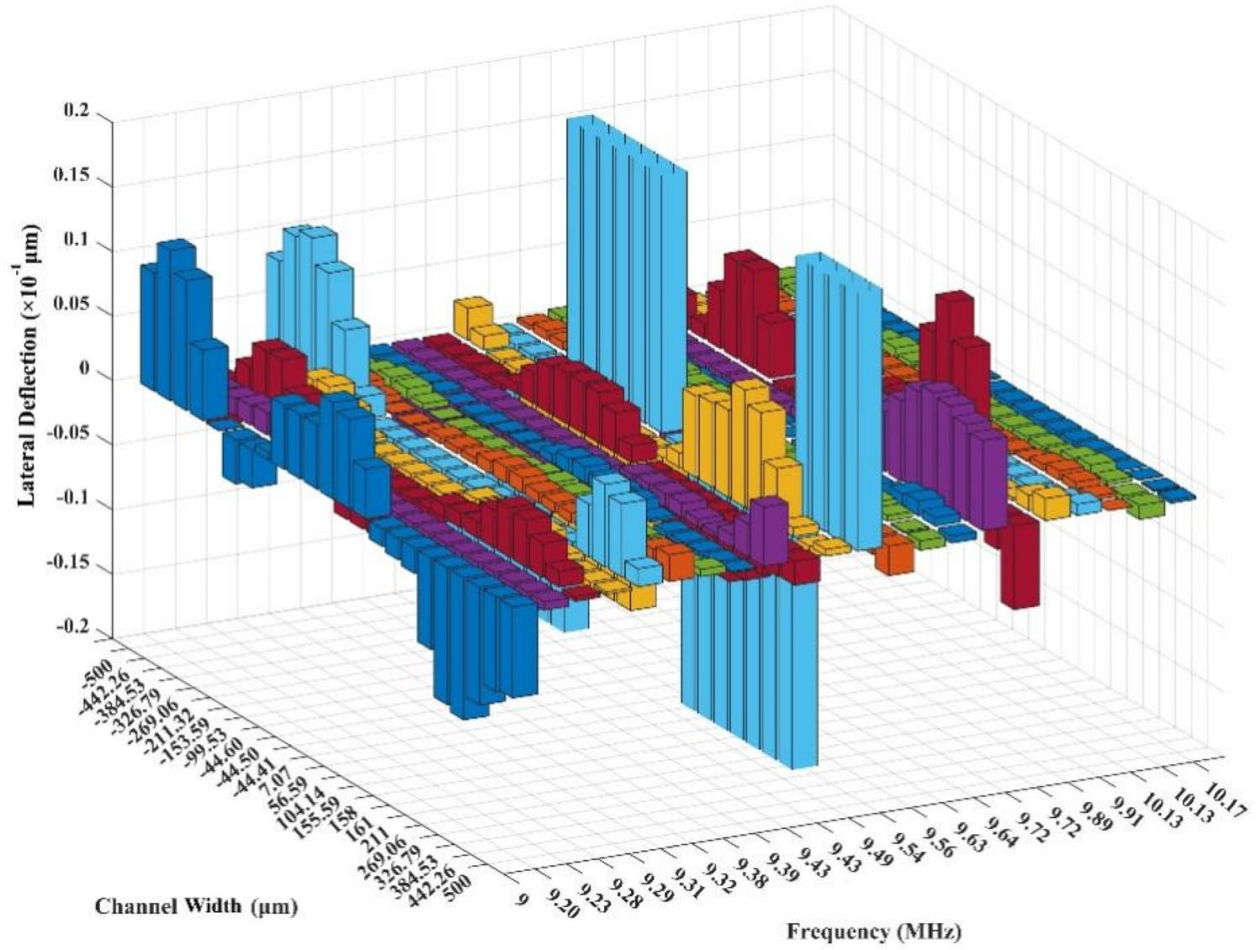
Harmonic analysis results for the substrate polarization amplitude along the z-axis in the derived frequencies from modal analysis.

The focus of the harmonic analysis is placed on two decisive factors. The first one is the amplitude of substrate displacement (polarization) along the z-axis in the microchannel place since it directly relates to the magnitude of ARF on the suspended particles in the fluid medium. The harmonic analysis results for the substrate displacement amplitude in the microchannel place along the dashed line (Fig. 1b) are presented in Fig. 2 for twenty-two mode shapes derived from modal analysis. Figure 2 shows the 9.54 MHz is the substrate's resonance frequency with about one μm polarization amplitude, which is about one hundred times higher than the average displacement of other frequencies.

**Figure 3:**
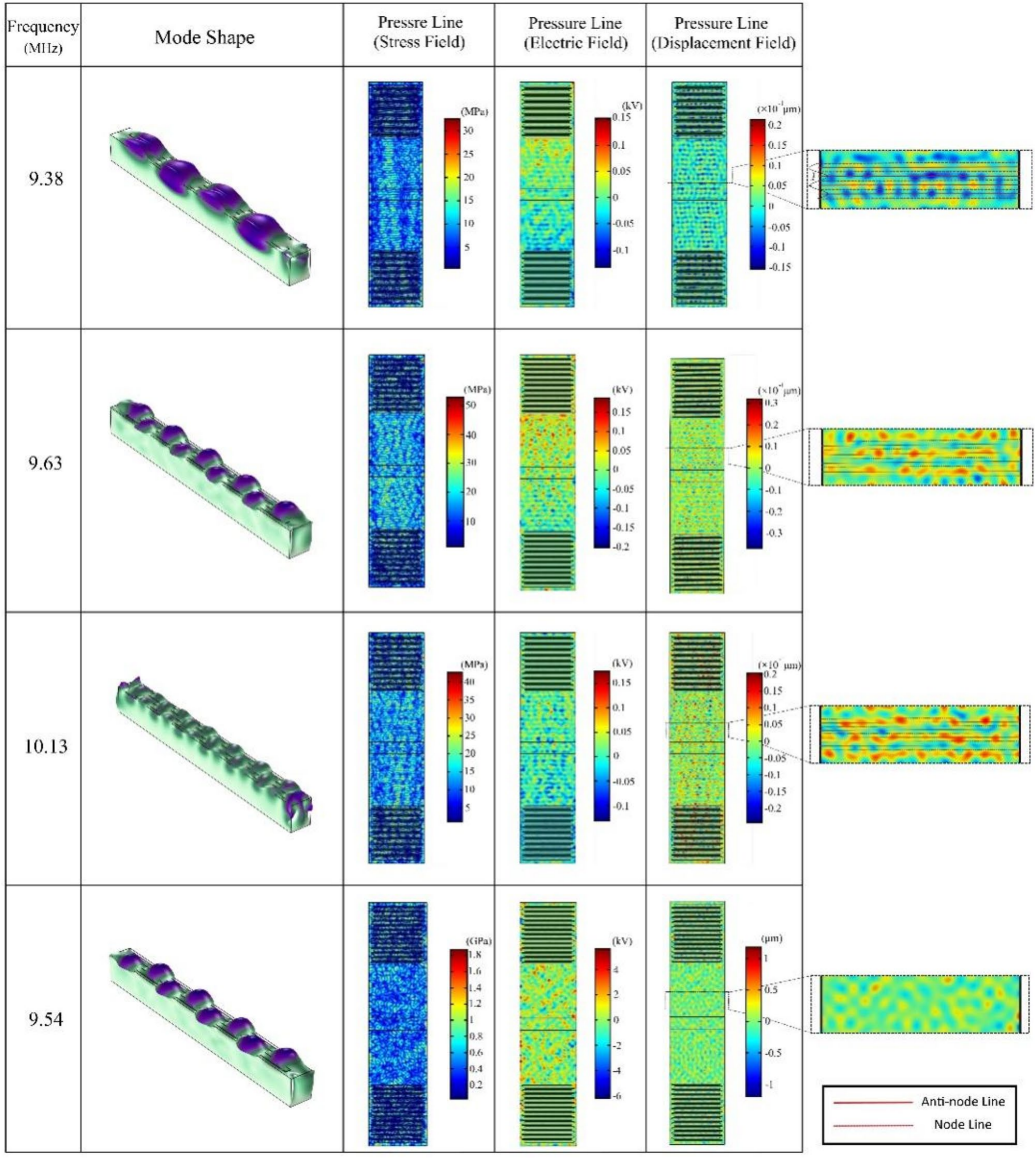
The analogy between frequencies of high contrast pressure lines and the resonance frequency of the substrate for selecting the optimal operating frequency.

The second one is the *quality* of the pressure lines, which is implied the perceptible formation of pressure lines, and as low as possible interference between them by considering stress, electric potential, and displacement field. Herein, quality is a fictive notion signifies the perceptible, decoupled occurrence of maximum (anti-nodes) and minimum (nodes) of the corresponding field along an assumed line on the substrate surface (Fig. 3). However, as shown in Fig. 3, fully-decoupled node and antinode lines would not be induced on the substrate due to the intrinsic anisotropy and electromechanical coupling of the piezoelectric material. Considering particle manipulation purpose, the importance of pressure line quality is more significant than the amplitude of the displacement field in opting for the optimal operating frequency. Since theoretical and experimental observations demonstrate that the nodes and anti-nodes of the induced standing acoustic field (pressure field) in the fluid medium lie immediately above the corresponding one on the substrate surface^43^. According to the harmonic analysis results, 9.38 MHz, 9.63 MHz, and 10.13 MHz frequencies would demonstrate pressure lines with the highest quality in the stress, electric potential, and displacement fields. To provide a better analogy, the simulated interference pattern in the mentioned fields along with corresponding vibration mode shapes for the mentioned frequencies and the resonance frequency of the substrate (9.54 MHz), are presented in Fig. 3. If the pressure lines were not of acceptable quality, the acoustic radiation force would provoke Brownian motion for the particles in the microchannel, which is not of interest in particle separation and focusing applications. Among frequencies that represent pressure lines with the highest quality, in the 9.63 MHz frequency, the highest displacement amplitude is induced in the substrate; thus, this frequency is utilized as the optimal actuation frequency.

### SSAW acoustofluidic model verification

**Figure 4:**
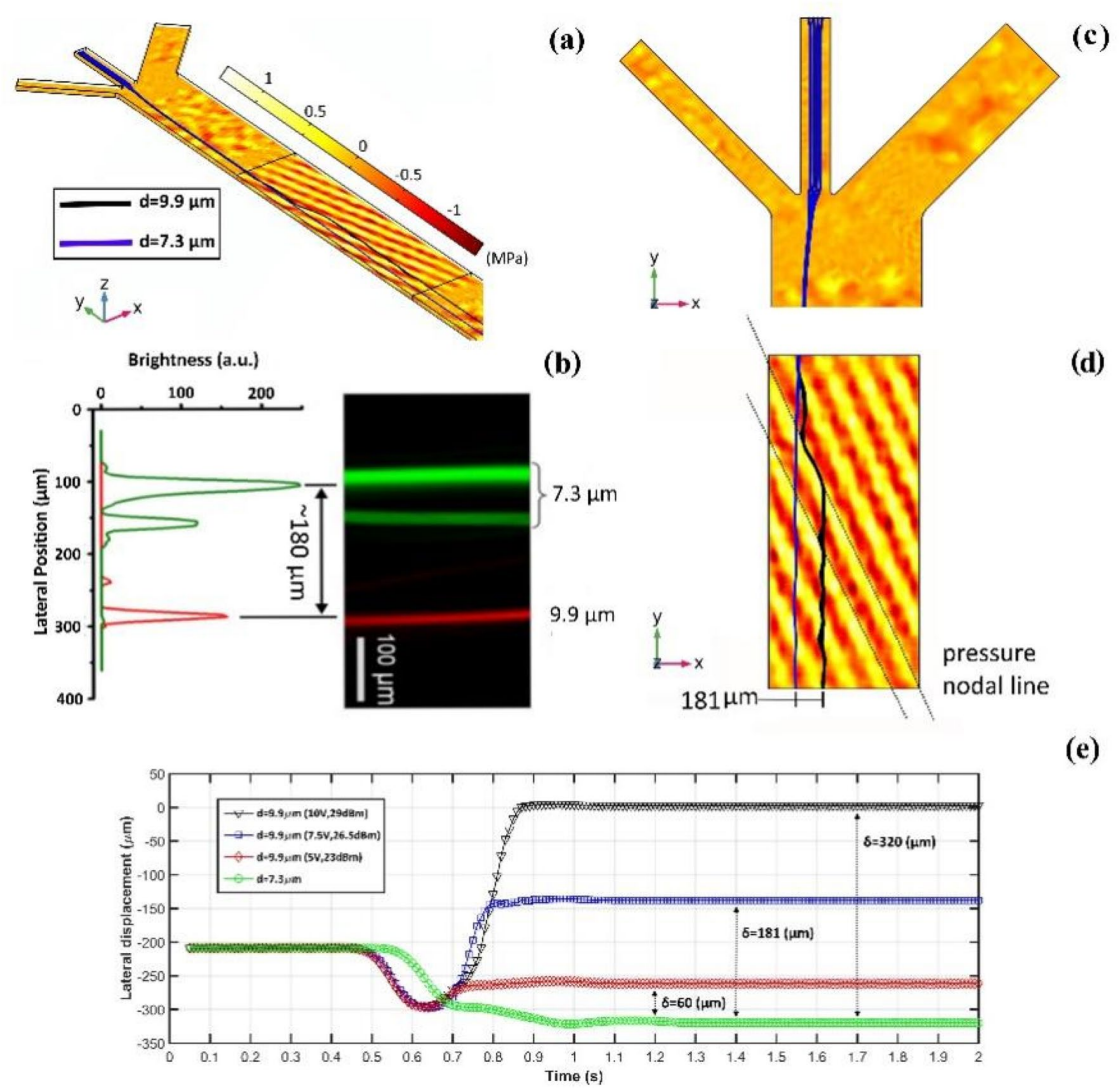
Simulation of PS beads separation with taSSAW field under 15° inclination angle, **(a)** the 3D first-order acoustic pressure field along with PS beads’ trajectories, **(b)** experimental investigation under simulated conditions in which the distribution profile of the PS beads on the working region departure is specified with fluorescence intensity adopted from ref 21, **(c)** the hydrodynamic focusing of particles with sheath flows on the xy plane, **(d)** the established diagonal pressure lines, movement of particles along with them, and interparticle distance after egress from working region, **(e)** lateral displacement of PS beads with 9.9 μm and 7.3 μm diameter vs. time along the width of the microchannel in different driving voltage (input power).

Figure 4 demonstrates the results of model verification for which, in line with Ding et al.^23^, the 19.32 MHz operational frequency (${\lambda }_{SAW}$ = 200 μm) besides 7.5 V driving voltage equivalent to 26.5 dBm input power is utilized. The procedure of calculating equivalent input power from the applied driving voltage in the presented simulation is explained in Appendix C as ESI. The established first-order acoustic pressure field and movement of polystyrene (PS) particles along with them in 3D is depicted in Fig. 4a. The dynamic processes of Fig. 4 in the conducted simulation can be seen in Movie S1 presented as ESI. Based on the reported experimental observations for the separation of PS beads with 9.9 μm and 7.3 μm diameters in a taSSAW device with 15° inclination angle of SSAW field working at 19.4 MHz frequency and 25 dBm input power, the maximum interparticle distance at the working region departure reaches to about 180 μm (Fig. 4b).

As shown in Fig. 4c, in conformity with the experimental setup, upstream hydrodynamic focusing with sheath flows is adopted for the presented simulation. The suspended particles would experience ARF (${{\varvec{F}}}^{rad}$ in Eq. 12), Stokes DF (${{\varvec{F}}}^{drag}$ in Eq. 14), inertial force, and buoyancy force upon entering the SSAW working region. In the presented simulation, due to the low height-to-width ratio (0.075) of the microchannel and assumed spherical geometry for the particles, the ARF and the buoyancy force on suspended microparticles are considered in balance with hydrodynamic DF created by particle–fluid relative velocity in the vertical direction. Therefore, the ARF and Stokes DF would dictate the particles’ trajectories. The ARF based on the acoustic contrast factor ($\varphi$) tends to accumulate particles at the pressure nodes or antinodes, which is given by^44^,

20 $$ \varphi (\kappa ,\rho ) = \frac{{5\rho_{p} - 2\rho_{f} }}{{2\rho_{p} + \rho_{f} }} - \frac{{\kappa_{p} }}{{\kappa_{f} }} $$

where $\rho$ and $\kappa$ stand for density and compressibility of the fluid (*f* subscript) and particle (*p* subscript), respectively. The PS beads’ acoustic contrast factor, based on Eq. (20) and presented physical properties in Table 1, would equal to 0.66; therefore, we expect that the PS beads in the SSAW field would confine at pressure nodal lines from a theoretical viewpoint.

Considering the simulated trajectory of particles presented in Fig. 4d, for the larger particles, ARF dominates over Stokes DF, and they migrate along pressure nodal lines coinciding with our theoretical expectation; however, for smaller particles, the dominancy of considered effective force is vice versa which leads to little lateral displacement. The simulated distribution of first-order acoustic pressure field in the taSSAW device (Fig. 4d) lying at a 15° inclination angle to the flow direction demonstrates the most significant advantages of considered on-chip configuration, which is that the particles traverse multiple neighboring pressure nodal lines along the path of the stream. This feature enables the device to capture the particles in subsequent neighboring nodal lines, providing that they escape from previous ones due to the stream conditions and intensity of the acoustic pressure field. Furthermore, as shown in Fig. 4d, through traversing each pressure line, maximum and minimum pressure anti-nodes repel particles until finally egress from the working region. These characteristics of the taSSAW devices, which are captured flawlessly with the presented 3D model, lead to their high separation efficiency and sensitivity compared to prevalent SSAW acoustofluidic devices^45,46^ in which the parallel pressure nodal lines to the flow direction restrain the maximum lateral displacement to ${\lambda }_{SAW}/4$. The variation of the particles’ lateral displacement with time along the microchannel width for 5 V, 7.5 V, and 10 V driving voltage equivalent to 23 dBm, 26.5 dBm, and 29 dBm input power, respectively, is demonstrated in Fig. 4e. Note that the applied input power and maximum lateral distance ($\delta$ in Fig. 4e) of the particles have a direct relationship, and with altering input power, the induced lateral displacement would be different. Considering Fig. 4, it can be discerned that, in approximately equal input powers, namely 25 dBm of the experimental setup and 26.5 dBm of the simulated device, the maximum lateral distance of particles reaches approximately 180 μm at the channel outlet. Consequently, the presented fully-coupled 3D model results coincide with the available experimental observation by a total error of 6.5%, which demonstrates the validity and accuracy of the presented model.

**Figure 5:**
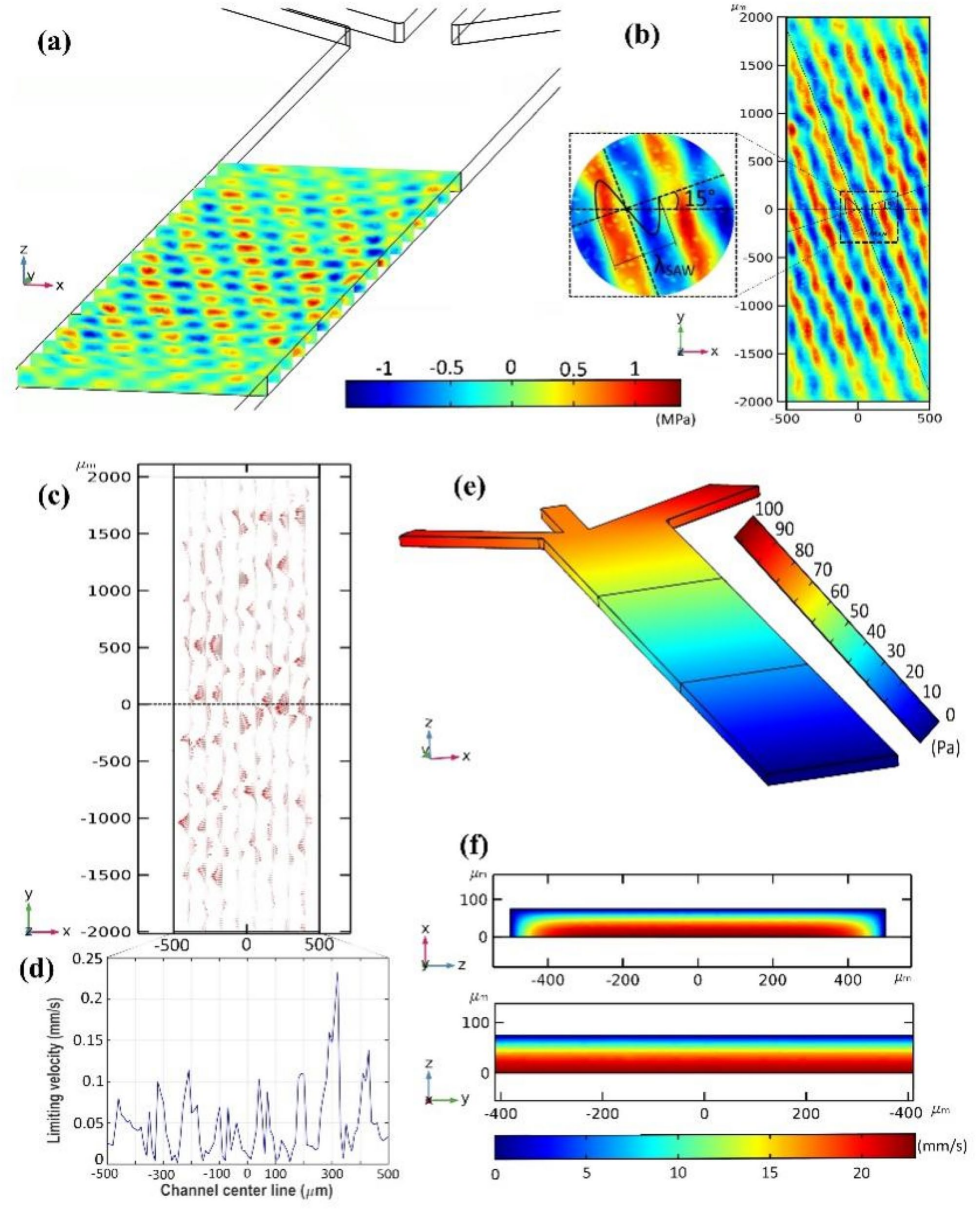
**(a)** First-order acoustic pressure field in the sequential sections of xz plane along the y-axis, **(b)** first-order acoustic pressure field in the xy plane with 20 μm height from the bottom of the channel along the z-axis, **(c)** the calculated limiting velocity field on the channel interface in the taSSAW working region, **(d)** the magnitude of limiting velocity along the horizontal center-line of the channel depicted with a dashed line in c **(e)** second-order pressure field of the microchannel, **(f)** second-order velocity field in the bulk of the fluid for xz and yz cross-sections.

To observe conciseness, the critical parameters of the conducted simulation are presented in Fig. 5 only for 7.5 V driving voltage (26.5 dBm input power). The first-order time-harmonic acoustic pressure field derived based on solving the Helmholtz equation (Eq. 8) in the fluid domain for the working region of taSSAW, is presented in Fig. 5a. The maximum absolute first-order acoustic pressure in the working region reaches 1 MPa, and the diagonal pattern of pressure nodal lines is simulated with acceptable accuracy in 3D (Fig. 5a), which is of utmost importance in specifying particle trajectories. The IDTs are in symmetry concerning the tilted vertical center-line of the microchannel (Fig. 5b); therefore, applied $\pi$ phase difference results in forming a full symmetric node and antinode about the tilted vertical symmetry line, and five ones along the whole channel width, which can be seen along the tilted horizontal dashed line in Fig. 5b. The limiting velocity field on the microchannel interface and the taSSAW working region, which was calculated based on Eq. (17) and presented codes in Appendix B, is depicted in Fig. 5c. The magnitude of limiting velocity along the horizontal center-line of the microchannel specified with the dashed line in Fig. 5c is presented in Fig. 5d. The maximum magnitude of the limiting velocity on the specified horizontal dashed line reaches 0.23 mm/s. The extremum points of the limiting velocity plot (Fig. 5d) contribute to the acoustic pressure nodes and anti-nodes in the established taSSAW field (Fig. 5b). With the utilization of derived limiting velocity as a slip boundary condition in the ‘creeping flow’ module of COMSOL Multiphysics, second-order pressure and velocity field are calculated in the bulk of the fluid domain presented in Fig. 5e,f, respectively. Note that the peak value of second-order pressure reaches 100 Pa, while the first-order pressure has a peak value of 1 MPa. This several order of magnitude scale-difference between time-harmonic and time-averaged quantities is one of the challenges of acoustofluidic processes’ simulation, leading to numerical accuracy problems. The first-order velocity field and second-order velocity field on the xy cross-section of the channel are presented in Fig. S5 as ESI.

### Sheathless particle separator utilizing taSSAW

**Figure 6:**
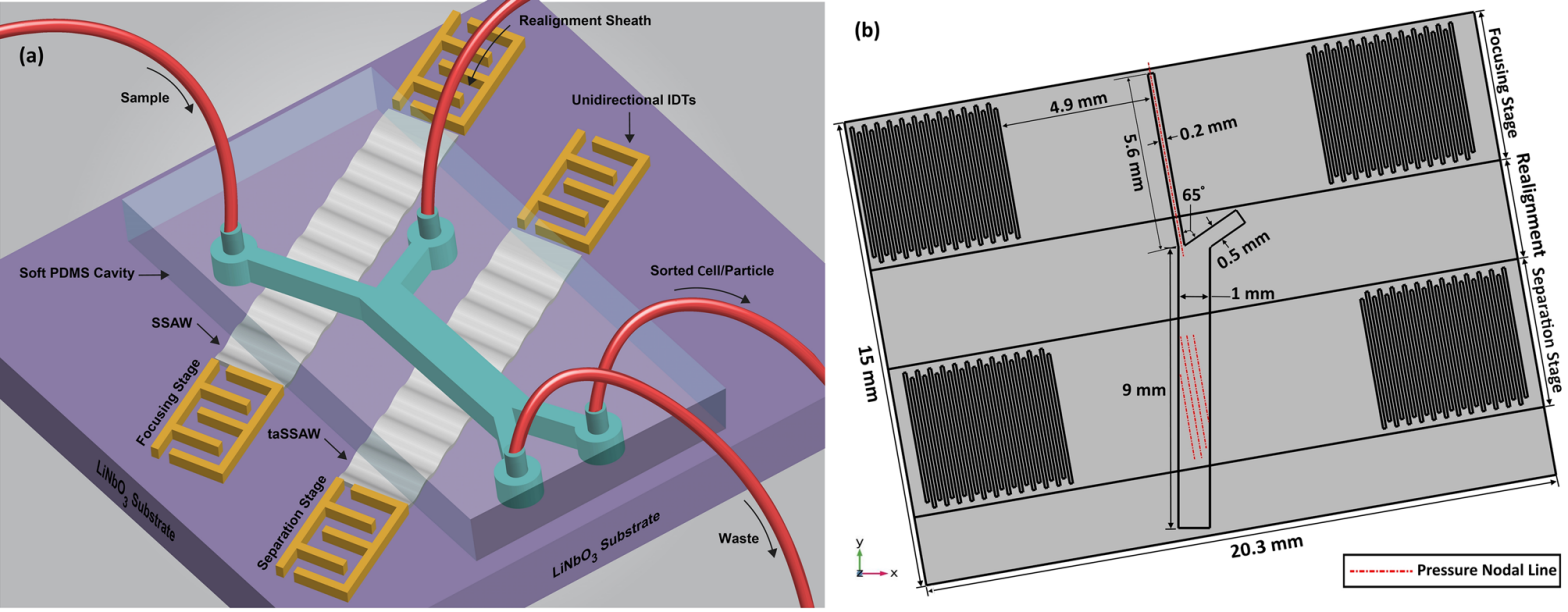
**(a)** Schematic illustration of the proposed sheathless particle separator platform using taSSAW, **(b)** the xy cross-section of the corresponding 3D FE model ($\uptheta$= 10°) in which detailed on-chip configuration, established pressure nodal lines, and different stages of the separation process are demonstrated.

In this section, as an application of the presented 3D FE model in further development and optimization of SSAW acoustofluidic devices, a semi-sheathless particle separator in which the enhanced characteristics of taSSAW configuration are harnessed as well is designed and simulated. To date, the design and fabrication of such on-chip configurations are just confined to conventional SSAW devices^47^. The conceptual design of a proposed on-chip configuration for a sheathless particle separator using taSSAW is articulated in Fig. 6a in which the whole separation process can be conducted in three stages: focusing, realignment, and separation. The advantages and justification of the proposed on-chip configuration are presented in Appendix C as ESI.

**Figure 7:**
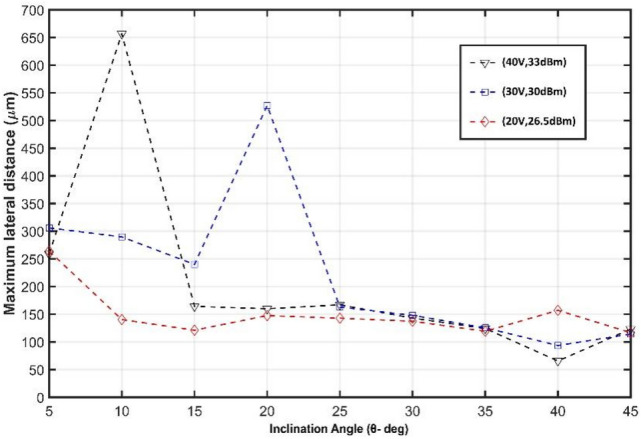
Optimization results of Inclination angle ($\theta$) to achieve the maximum feasible separation efficiency for PS particles of 4 μm and 10 μm diameter.

The potential of the proposed sheathless platform in the separation of PS microbeads with 4 μm and 10 μm diameter, and, subsequently as a biomedical-biological application, in the sorting of MCF-7 human breast cancer cell (20 μm diameter) from normal leukocytes (WBCs) (12 μm diameter) are investigated with verified and developed 3D FE model. According to the conducted modal and harmonic analysis, ${\lambda }_{SAW}$= 400 μm (leading to 9.63 MHz operational frequency) besides inlet flow velocity of 6 mm/s are considered. In the focusing stage, the microchannel width is considered ${\lambda }_{SAW}/2$ to encompass only one pressure nodal line. The deposited IDTs and substrate should be rotated concerning the flow direction at an inclination angle ($\theta$ in Fig. 1d) to establish the taSSAW field in the separation stage. This angle is specified through an optimization process elaborated in Appendix C as ESI with the developed 3D numerical model to achieve the highest feasible separation efficiency by considering different driving voltages (input powers). In the conducted optimization, the inclination angle ($\theta$) is variated from 5° to 45° for three different driving voltages (input powers), including 20 V (26.5 dBm), 30 V (30 dBm), and 40 V (33 dBm). In each one, the maximum separation inter-particle distance after egressing the working region is determined, for which the captured results are presented in Fig. 7.

Based on the optimization results, 40 V (33 dBm) driving voltage under $\theta$=10° is utilized to separate PS beads in the simulation of the proposed sheathless configuration, and presented geometrical dimensions in Fig. 6b are based on this optimum state. For the considered cells, we suffice to the conducted inclination angle optimization in the literature, and 30 V (24.7 dBm) driving voltage under $\theta$=10° is adopted here^23^.

The particles’ inlet of the microchannel should be inclined as well at an angle identical with $\theta$ (Fig. 6) to make the induced pressure nodal line parallel with the flow direction in the focusing zone, i.e., the established acoustic field in the first stage for particle focusing should be conventional SSAW. Nevertheless, the egressing of particle-laden flow from the focusing zone at an inclination angle parallel to the induced pressure nodal lines (deposited IDTs) would conflict with the underlying mechanism of taSSAW configuration in improving separation efficiency attained by traversing multiple pressure nodal lines along the flow direction. Furthermore, it can engender clogging problems and aggregation of particles along the sidewall of the microchannel. Thus, to overcome the mentioned challenges, it is requisite to introduce a side flow just after the focusing stage by which the direction of particle-laden flow would become parallel to the microchannel sidewall, namely the realignment stage (Fig. 6).

**Figure 8:**
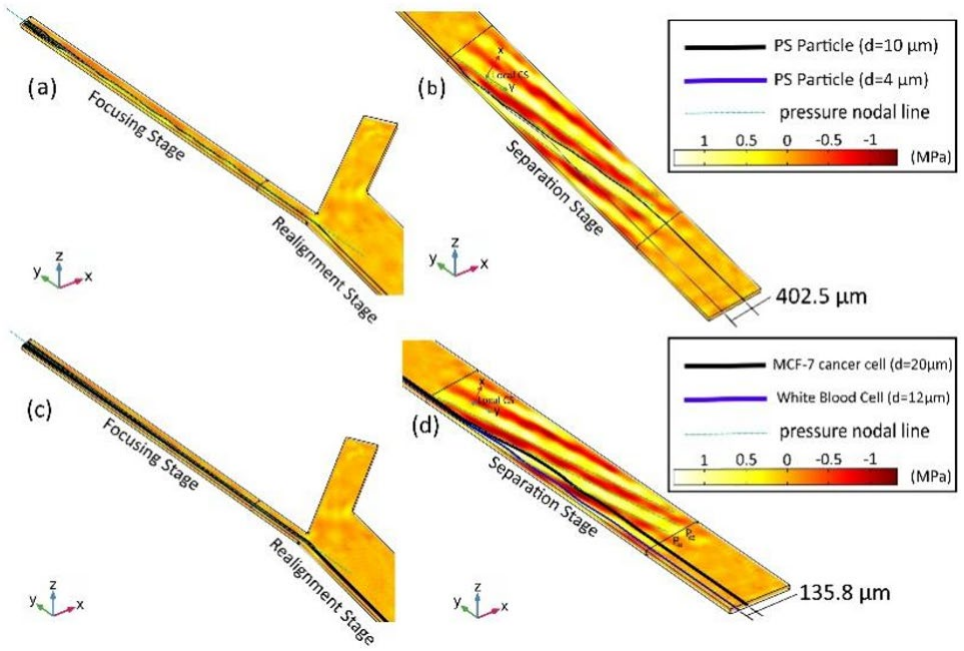
The isometric 3D view of microchannel and particle trajectories, focusing and realignment stage of **(a)** PS particles (10 V driving voltage), and **(c)** WBCs and MCF-7 cancer cells (5 V driving voltage), separation stage of **(b)** PS particles (40 V,$\theta$ = 10°), and **(d)** WBCs and MCF-7 cancer cells (30 V,$\theta$ = 10°).

The simulation results of the proposed sheathless platform by considering the whole three stages are presented in Fig. 8 for PS particles (Fig. 8a,b), and considered cells (Fig. 8c,d), which is including established pressure nodal lines, first-order acoustic pressure distribution, and the particles’/cells’ trajectory. The dynamic processes of Fig. 8 in the conducted simulation can be seen in Movie S2, and Movie S3 presented as ESI.

Based on the presented physical properties in Table 1, the acoustic contrast factor ($\varphi$) of WBCs and MCF-7 cancer cells suspended in an aqueous solution would be equal to 0.13, according to Eq. (20). Therefore, from a theoretical viewpoint, we expect that the considered cells similar to PS beads in the established SSAW field would accumulate along the pressure nodal lines and be repelled by the extremum acoustic pressure anti-nodes. As demonstrated in Fig. 8, the simulated particles’/cells’ trajectories coincide well with the explained theoretical expectations. As shown in Fig. 8a,c, with adopting appropriate geometrical dimensions in the designing process, single pressure nodal line is induced in the focusing stage, which attracts both PS beads, and WBCs and MCF-7 cancer cells to itself due to the provoked ARF in the established SSAW field. The relatively smaller width of the working region in the focusing stage compared to the separation one demands a lower input power level (10 V driving voltage), which leads to the decrement of the maximum absolute value of the first-order acoustic pressure field, which is approximately 0.5 MPa in the focusing stage (Fig. S9 in ESI). Once the focused particles enter the realignment stage, the introduced side flow with an initial flow velocity of 18 mm/s would shoulder the responsibility of redirecting the particle-/cell-laden flow stream such that it would become parallel to the sidewall of the microchannel. In the taSSAW working region for both PS particles and considered cells, the maximum value of the first-order acoustic pressure field reaches 1 MPa. The inclined pressure nodal lines pattern is derived with acceptable accuracy, which is highly essential in dictating particle trajectories. As shown in Fig. 8b,d , the maximum lateral inter-particle/-cell distance at the channel outlet reaches 402.5 μm and 135.8 μm, respectively. The lateral displacement of PS particles and considered cells during the simulation process are demonstrated in Fig. S10 presented as ESI.

As pointed out before, the taSSAW on-chip configuration compared to the conventional SSAW device can enhance the device's separation efficiency and sensitivity through two mechanisms. The first procedure is the particles’ movement along the inclined pressure nodal lines, which can be observed in Fig. 8b,d for both PS particles and considered cells. The second procedure is trapping in successive inclined pressure nodal lines along the flow direction, seen in Fig. 8d during the cell separation process. Once the MCF-7 cancer cells (black path in Fig. 8d) traverse the P_0-1_ nodal line (about 1.2 s in Fig. S10b), they trap along the subsequent P_0-2_ nodal line (about 1.3 s in Fig. S10b) and finally egress from the working region. Thus, the presented simulation procedure can capture both underlying improved separation mechanisms of the taSSAW configuration in a 3D manner, which might occur individually or simultaneously depending on the simulated chip conditions.

**Figure 9:**
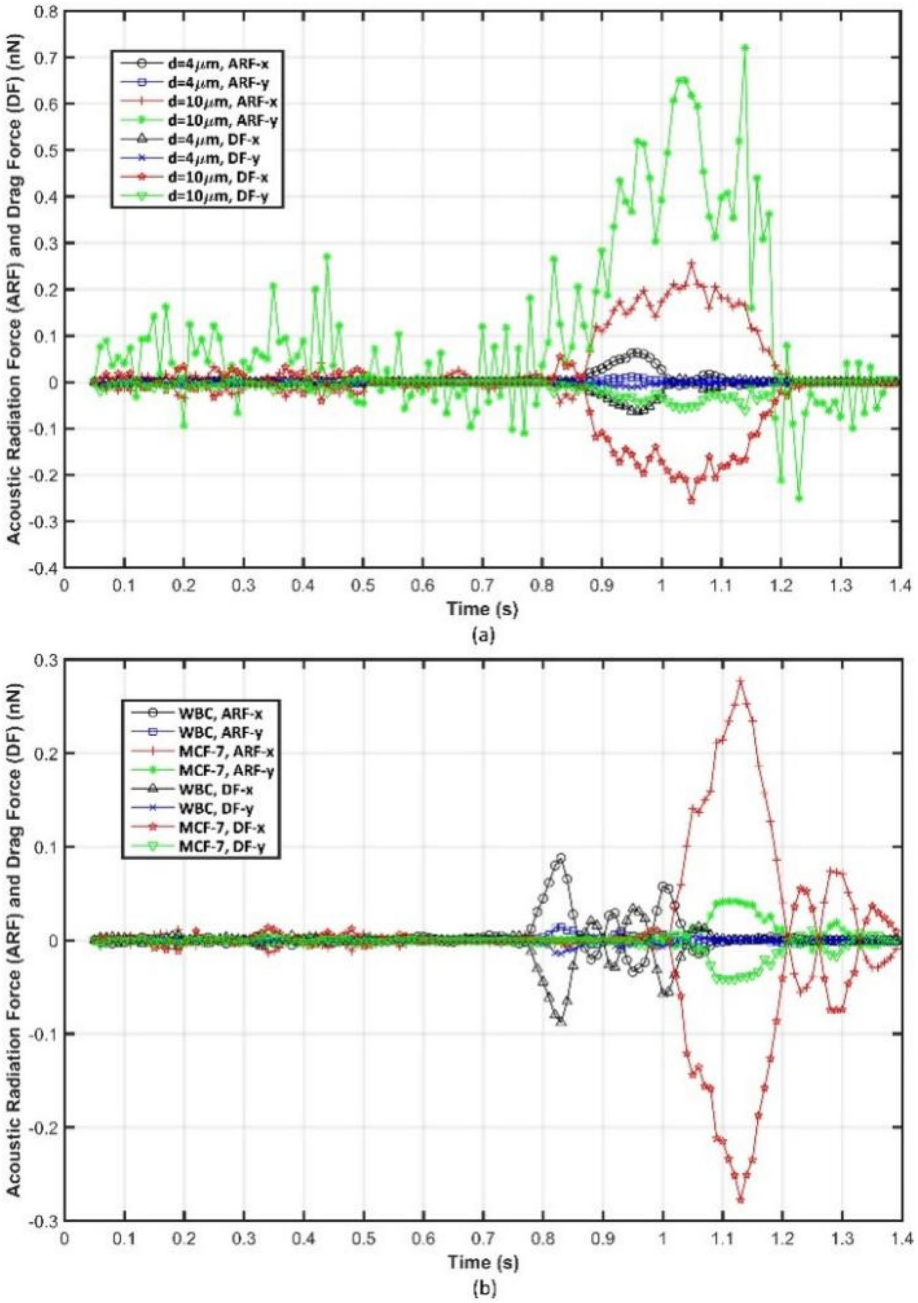
The applied acoustic radiation force and Stokes drag force on **(a)** the PS particles, and **(b)** WBCs and MCF-7 cancer cells.

For the sake of brevity, the second-order time-averaged pressure and velocity field in the bulk of the fluid domain would be presented just for PS particles in Fig. S11 as ESI. The ARF and Stokes DF on the PS particles (Fig. 9a) and considered cells (Fig. 9b), which were calculated based on Eqs. (12) and Eq. (13), and presented codes in Appendix B, are presented in Fig. 9.

The depicted forces in Fig. 9 are calculated in the local coordinate system (Fig. 8), and the ARF and Stokes DF on the same particle/cell and in the same direction is demonstrated with an identical color to facilitate the analogy. As shown in Fig. 9, and conformity with our theoretical expectations, the applied ARF on the larger particle/cell (10 μm PS particle and MCF-7 cancer cell) is more intense than the smaller ones (4 μm PS particle and WBC); since it has a cubic dependence on the particle’s/cell’s radius based on Eq. (12). The more intense ARF leads to the higher relative velocity between particle and fluid flow, which would increase the Stokes DF on the suspended particles based on Eq. (14). As shown in Fig. 9, the ARF and Stokes DF are in equilibrium with each other in all directions except the local y-axis (parallel to the established nodal lines (Fig. 8)) for the larger PS particles, and it can elucidate the reasons behind the approximately undeviating displacement of them along the initially trapped in nodal line once entering the separation zone as depicted in Fig. 8b. The dominance of ARF over Stokes DF would lead to the larger PS particles' accelerated motion along the local y-axis. Thus, the less intense ARF on the local x-axis (perpendicular to the pressure nodal lines (Fig. 8)) could not alter their trajectory until near the end of the separation stage (about 1.1 s in Fig. 9a) in which due to an abrupt reduction in the y-axis ARF, the ARF along the x-axis could change the trajectory. Thenceforth, the y-axis ARF fluctuations (Fig. 9a) could not entirely modify the larger PS particles’ trajectory due to the preceding attained momentum by them, which is intensified the inertia effect on preserving their path.

On the other hand, As shown in Fig. 9b, analysis of the influential forces on the considered cells demonstrates that the applied ARF, and consequently induced Stokes DF, on the cells along the x-axis of the local coordinate system is more intense than the ones applied along the local y-axis, and ARF and Stokes DF on the cells are in equilibrium with each other in both local directions. The results of the presented simulation in Fig. 9 demonstrate even though ARF and Stokes DF on the same particle and in the same direction mostly are in balance with each other, under particular circumstances, the aforementioned equilibrium would not be satisfied. Therefore, an accurate prior evaluation of the effective parameters on the operation of the investigated acoustofluidic device based on the presented 3D simulation procedure or experimental observations is mandatory to determine whether the mentioned equilibrium is an eligible assumption for the 2D simplification of the device. An analogy between the magnitude of applied ARF on the PS particles and considered cells besides analysis of the influential parameters on ARF during the conducted simulation are presented in Fig. S12 as ESI.

## Conclusion

The 3D numerical simulation of boundary-driven acoustic streaming in the acoustofluidic devices utilizing SSAWs has been conducted based on the limiting velocity finite element method, and its influences on the microparticles’ acoustophoretic motion are investigated. Through this efficient computational method based on a robust scientific basis, subtle simulation considerations for different aspects of a real acousto-microfluidic device are elaborated. The discussion dominantly investigates SSAW propagation in the piezoelectric substrate through modal and harmonic analysis, fluid-substrate interactions, the particles' acoustophoretic motion, required boundary conditions, meshing technique, and demanding computational cost. Despite valuable provided insight of 2D simulations in acoustofluidic problems, the developed 3D model can overcome their limitations in capturing the impression of established SSAW and taSSAW field non-uniformities along the channel length on the particle trajectory, and final inter-particle distance at channel outlet under highly interactive involved parameters. The presented 3D model would have sufficient capability and versatility to be adopted to simulate novel complex on-chip configurations like taSSAW microfluidic devices^23^ and disposable acoustofluidic chips^48^. Furthermore, it helps to make a more accurate prior approximation for 2D simplification of the favorable device, which might not always be straightforward.

As an experimental validation of our numerical simulation, a taSSAW platform (13 mm $\times$ 19.6 mm $\times$ 0.5 mm) for separating PS particles of 7.3 μm and 9.9 μm diameter under identical conditions is simulated, which demonstrates acceptable agreement with reported experimental observations. Subsequently, as an application of the presented 3D model in further the design and optimization of the acoustofluidic devices, a novel sheathless taSSAW platform (15 mm $\times$ 20.3 mm $\times$ 0.5 mm) for the separation of PS particles (4 μm and 10 μm), and WBCs from MCF-7 cancer cells (12 μm and 20 μm diameter) is conceptualized and designed. In this way, the model’s capability and versatility in examining device features such as optimization inclination angle, determining inlet flow velocities, calculating limiting velocity, ARF, Stokes DF, first-order, and second-order quantities, and evaluating the eligibility of 2D simplification are discussed. In summary, the obtained results demonstrate the presented 3D fully-coupled model, due to the more time-/cost-efficient performance than precedented 3D models, the capability to model complex on-chip configurations, and overcome shortcomings and limitations of 2D simulations could be considered as a powerful tool in further the designing and optimizing SSAW microfluidics. However, the utilized semi-analytical limiting velocity approach has its restrictions, including larger boundaries’ curvature in comparison to the viscose boundary layer thickness, strictly laminar flow regime, and established streaming field with high Womersley number ($\left|M\right|\gg 1$), which should be considered before applying it on a high-frequency SAW system^16,49^.

## Supplementary Information

Supplementary Information.

Supplementary Movie S1.

Supplementary Movie S2.

Supplementary Movie S3.
