# Supplementary material for: 3D numerical simulation of acoustophoretic motion induced by boundary-driven acoustic streaming in standing surface acoustic wave microfluidics
Source: Sci Rep. 2021 Jun 25;11:13326. doi: 10.1038/s41598-021-90825-z (PMC8233446; doi:10.1038/s41598-021-90825-z)
Supplement: Supplementary file 1 — Supplementary Information. [file 41598_2021_90825_MOESM1_ESM.pdf]

Supplementary Information

3D Numerical Simulation of Acoustophoretic Motion Induced by Boundary-Driven Acoustic Streaming in Standing Surface Acoustic Wave Microfluidics

Mohammad Sadegh Namnabat, Mahdi Moghimi Zand*, and Ehsan Houshfar

Appendix A. Investigating Lithium Niobate Physical Properties for Different Crystal Cuts

Lithium Niobate ($LiNbO_3$) is a compound of Niobium, Lithium, and Oxygen, not occurring naturally in mostly demanded monocrystalline form. It serves in various device applications due to the favorable elastic, piezoelectric, dielectric, acousto-optical, electro-optical, pyroelectric, photoelectric, and photovoltaic properties, precisely, an excellent piezoelectric coupling factor and very high Curie temperature making it a prime candidate for ultrasonic device applications. Lithium Niobate possesses several useful cuts extensively used in transducer applications, e.g., YZ $LiNbO_3$ (i.e., Y-axis crystal cut, Z-axis propagation) or 128° rotated YX $LiNbO_3$. The monocrystals would be sawed by manufacturers depending on the required cut for a particular application (1).

The physical properties of Lithium Niobate are presented in the literature for fundamental crystal cut (Z-cut $LiNbO_3$) with trigonal (class, 3m) crystal system, which shows six independent elastic-stiffness coefficients; thus, in Voigt (contradicted) notation, the elasticity tensor ($\boldsymbol{c} = c_{IK}$) is given by,

$$\left[c_{ij}\right] = \begin{bmatrix} c_{11} & c_{12} & c_{13} & c_{14} & 0 & 0 \\ c_{12} & c_{11} & c_{13} & -c_{14} & 0 & 0 \\ c_{13} & c_{13} & c_{33} & 0 & 0 & 0 \\ c_{14} & -c_{14} & 0 & c_{44} & 0 & 0 \\ 0 & 0 & 0 & 0 & c_{44} & c_{14} \\ 0 & 0 & 0 & 0 & c_{14} & c_{66} \end{bmatrix} \tag{S1}$$

Where $c_{66} = {c_{11} - c_{12}}/{2}$. The piezoelectric tensor ($\boldsymbol{e} = e_{IK}$) of such crystals would constitute four independent coefficients represented as follows in Voigt notation.

$$\left[e_{ijk}\right] = \begin{bmatrix} 0 & 0 & 0 & 0 & e_{15} & -e_{22} \\ -e_{22} & e_{22} & 0 & e_{15} & 0 & 0 \\ e_{31} & e_{31} & e_{33} & 0 & 0 & 0 \end{bmatrix} \tag{S2}$$

And they show two independent dielectric coefficients ($\boldsymbol{K} = \left(K_{ij}\right)$) or relative permittivity ($\boldsymbol{\varepsilon_r} = \boldsymbol{\varepsilon}/\varepsilon_0$ in which $\boldsymbol{\varepsilon_r}$, $\boldsymbol{\varepsilon}$, and ε_0 are relative permittivity, absolute permittivity, and vacuum permittivity, respectively.).

$$[K_{ij}] = \begin{bmatrix} K_{11} & 0 & 0 \\ 0 & K_{11} & 0 \\ 0 & 0 & K_{33} \end{bmatrix} \quad \text{(S3)}$$

Determining these twelve independent coefficients is an arduous task for which the presented ones by Ledbetter et al. (2) would be utilized here, brought in Table 1.

Table 1. The independent coefficients of elasticity tensor (GPa), piezoelectric tensor (C/m^2), and dielectric tensor for fundamental crystallographic cut (Z-cut) of $LiNbO_3$

c_{11}	c_{12}	c_{13}	c_{14}	c_{33}	c_{44}	e_{15}	e_{22}	e_{31}	e_{33}	K_{11}	K_{33}
199.5	55.27	67.67	8.7	235.2	59.48	3.65	2.39	0.31	1.72	45.05	26.2

The physical properties of $LiNbO_3$ in other crystal cuts would be determined through a method known as coordinate transformation, explained by Auld (3). To conduct the tensor transformation procedure, a crystallographic fundamental orthogonal coordinate system (CS) should be defined on the hexagonal unit cell of $LiNbO_3$ defined by (a_1, a_2, a_3, c) Miller indices for which, as depicted in Fig. S1, X-axis is along a_1, Z-axis is collinear with c, and Y-axis is determined with the right-hand rule. The relationship between the fundamental CS (X, Y, Z) and a given crystal cut's CS (x_1, y_1, z_1) is specified with Euler's angles (φ, θ, ψ) defined as three successive rotation of fundamental CS around (Z, X', Z'')-axes (Fig. S1). The corresponding Euler' angles to different crystallographic cuts are presented in Table 2.

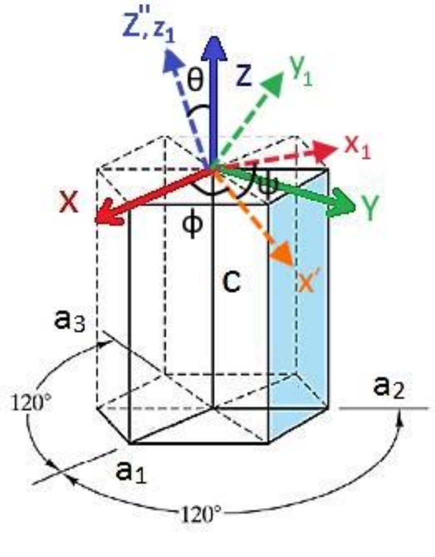

Figure S 1. The crystallographic fundamental orthogonal CS defined on hexagonal unit cell of $LiNbO_3$, and successive rotations under Euler's angles

Table 2. The corresponding Euler's angles to different crystallographic cuts of $LiNbO_3$

Euler's angles (°)	X-cut	Y-cut	Z-cut	XY α°	XZ α°	YX α°	YZ α°	ZX α°	ZY α°
ϕ	0	0	0	0	0	0	0	0	0
θ	90	90	0	90+ α	90	α-90	90	-α	α
ψ	90	0	0	90	90- α	0	- α	0	90

By following the explained procedure in (3), a MATLAB code is developed to calculate tensor parameters of required $LiNbO_3$ physical properties in other crystallographic configuration. According to Table 2, the equivalent Euler's angles to YX 128 crystal cut of $LiNbO_3$ are (0°, 38°, 0°) for which the calculated physical properties, utilized in the COMSOL Multiphysics for the presented numerical simulation, are as follows.

$$\left[c_{ij}\right]=\begin{bmatrix} 2 & 0.68 & 0.55 & 0.08 & 0 & 0 \\ 0.68 & 1.88 & 0.84 & 0.08 & 0 & 0 \\ 0.55 & 0.84 & 2.14 & 0.07 & 0 & 0 \\ 0.08 & 0.08 & 0.07 & 0.76 & 0 & 0 \\ 0 & 0 & 0 & 0 & 0.68 & -0.07 \\ 0 & 0 & 0 & 0 & -0.07 & 0.56 \end{bmatrix}\times 10^{11}\left({N}/{m^2}\right) \quad \text{(S4)}$$

$$\left[e_{ijk}\right]=\begin{bmatrix} 0 & 0 & 0 & 0 & 1.35 & -4.36 \\ -1.69 & 4.48 & -1.55 & 0.2 & 0 & 0 \\ 1.72 & -2.43 & 2.57 & 0.71 & 0 & 0 \end{bmatrix}\left({C}/{m^2}\right) \quad \text{(S5)}$$

$$\left[k_{ij}\right]=\begin{bmatrix} 45.05 & 0 & 0 \\ 0 & 37.91 & -8.15 \\ 0 & -8.15 & 33.35 \end{bmatrix} \quad \text{(S6)}$$

Appendix B. Numerical Expressions

Here, the calculation of limiting velocity and acoustic radiation force numerical expressions based on the internal functions and variables of COMSOL Multiphysics 5.3a would be investigated. For the definition of individual expressions, please refer to the COMSOL Multiphysics documentation (4).

Acoustic Streaming by the limiting velocity method

The boundary-driven acoustic streaming in the bulk of the fluid can be calculated with the limiting velocity finite element method. As explained in section 3, this method is based on an analytical solution of the viscose boundary layer and calculating the second-order slip velocity just outside the boundary layer vortices. The following expressions, adopted and modified from Hahn Ph.D.

thesis (5), allow calculating the limiting velocity in a 3D fluid cavity while the structural vibrations of cavity walls are taken into account.

Note that the limiting velocity would be calculated based on Eq. 13 and Eq. 14 in the local CS on the fluid-substrate interface, while the required first-order fluid and substrate velocities as well as utilized slip velocity in the COMSOL Multiphysics for solving 'creeping flow' physics are in the global CS. Therefore, coordinate transformation for the calculation, and then utilization of the limiting velocity would be indispensable.

The fluid-substrate relative velocities ($u1$, $v1$, and $w1$ in the X, Y, and Z global CS, respectively) would be calculated as,

```
u1 = acpr.vx-solid.u_tX
v1 = acpr.vy-solid.u_tY
w1 = acpr.vz-solid.u_tZ
```
(S7)

Then, a coordinate transformation from global CS to the local CS on the fluid-substrate interface with corresponding unit vectors should be conducted. The tangential directions in the local CS are denoted by $t1$ and $t2$, and the normal direction on the interface is denoted by n. The fluid-substrate relative velocities in the tangential interface directions would be $ut1$ and $ut2$, and the normal substrate velocity would be stated as un. For coordinate transformation, we have,

```
ut1 = (u1*t1X)+(v1*t1Y)+(w1*t1Z)
ut2 = (u1*t2X)+(v1*t2Y)+(w1*t2Z)
un = (acpr.vx*nx)+(acpr.vy*ny)+(acpr.vz*nz)
```
(S8)

Then, the complex conjugate of local relative velocities should be calculated.

```
ut1c = conj(ut1)
ut2c = conj(ut2)
unc = conj(un)
```
(S9)

With the calculated local relative velocities, and based on the Eq. 13 and Eq. 14, the limiting velocity in the local CS would be calculated. Before presenting numerical expressions, it is beneficial to review the directional derivative of a vector field $f(x)$ along a given vector v,

$$\nabla_v f(x) = \nabla f(x).v \tag{S10}$$

Therefore, about the derivative of the local relative velocity $ut1$ along the tangential direction $t1$ we have,

$$\frac{dut1}{dt1} = \nabla ut1.t1 = \frac{dut1}{dY}t1X + \frac{dut1}{dY}t1Y + \frac{dut1}{dZ}t1Z \tag{S11}$$

By calculating other derivatives in Eq. 13 and Eq. 14 with an analogous procedure, the local limiting velocities $ult1$ and $ult2$ in the interface tangential directions would be,

ult1 = -1/(4*omega)*real(ut1*(d(ut1c,x)*t1X+d(ut1c,y)*t1Y
+d(ut1c,z)*t1Z)+ut2*(d(ut1c,x)*t2X+d(ut1c,y)*t2Y+d(ut1c,z)
t2Z)+ut1c((2+i)*((d(ut1,x)*t1X+d(ut1,y)*t1Y+d(ut1,z)
*t1Z)+(d(ut2,x)*t2X+d(ut2,y)*t2Y+d(ut2,z)*t2Z)+(d(un,x)
*nx+d(un,y)*ny+d(un,z)*nz))-(2+3*i)*(d(un,x)*nx+d(un,y)
*ny+d(un,z)*nz))) (S12)

ult2 = -1/(4*omega)*real(ut1*(d(ut2c,x)*t1X+d(ut2c,y)*t1Y
+d(ut2c,z)*t1Z)+ut2*(d(ut2c,x)*t2X+d(ut2c,y)*t2Y+d(ut2c,z)
t2Z)+ut2c((2+i)*((d(ut1,x)*t1X+d(ut1,y)*t1Y+d(ut1,z)
*t1Z)+(d(ut2,x)*t2X+d(ut2,y)*t2Y+d(ut2,z)*t2Z)+(d(un,x)
*nx+d(un,y)*ny+d(un,z)*nz))-(2+3*i)*(d(un,x)*nx+d(un,y)
*ny+d(un,z)*nz))) (S13)

Then, the limiting velocities $ult1$ and $ult2$ in the local CS should be transferred to the global CS, and the calculated limiting velocities ul, vl, and wl could be utilized as slip boundary condition in 'creeping flow' simulation.

ul = ult1*t1X+ult2*t2X
vl = ult1*t1Y+ult2*t2Y (S14)
wl = ult1*t1Z+ult2*t2Z

Acoustic Radiation Force

The capability of COMSOL Multiphysics in defining a general external force based on internal functions and variables should be utilized to calculate time-averaged second-order acoustic radiation force on a suspended particle in a viscose fluid according to Eq. 19 to Eq. 22 since a predefined node for applying complicated acoustic force is not anticipated in this software. The acoustic radiation force would be calculated in the global CS, and then transferred to the local CS for the utilization in the 'particle tracing' simulation. As depicted in Fig. S2, the local y-axis is parallel to the established pressure nodal lines while the local x-axis is perpendicular to them.

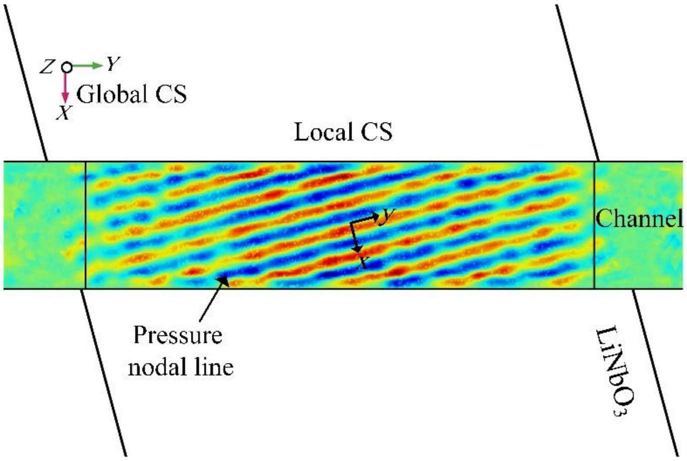

Figure S 2. Local CS and global CS utilized for calculating acoustic radiation force on suspended particles

Based on the Eq. 19, the acoustic radiation force numerical expression can be separated to pressure gradient (PRF) and velocity gradient (VRF) parts.

$$PRF = p_1^* \nabla p_1 \text{ and } VRF = v_1^* \nabla v_1 \tag{S15}$$

Then, the PRF and VRF terms would be calculated based on the first-order acoustic quantities, and Eq. 19 to Eq. 22 in the global CS as follows.

PRF_x = conj(acpr.p_t)*acpr.gradpx
PRF_y = conj(acpr.p_t)*acpr.gradpy (S16)
PRF_z = conj(acpr.p_t)*acpr.gradpz

VRF_x = conj(acpr.vx)*(-acpr.hessianpxx/(acpr.iomega*rho_w))
+conj(acpr.vy)*(-acpr.hessianpxy/(acpr.iomega*rho_w))
+conj(acpr.vz)*(-acpr.hessianpxz/(acpr.iomega*rho_w))
VRF_y = conj(acpr.vx)*(-acpr.hessianpyx/(acpr.iomega*rho_w))
+conj(acpr.vy)*(-acpr.hessianpyy/(acpr.iomega*rho_w)) (S17)
+conj(acpr.vz)*(-acpr.hessianpyz/(acpr.iomega*rho_w))
VRF_z = conj(acpr.vx)*(-acpr.hessianpzx/(acpr.iomega*rho_w))
+conj(acpr.vy)*(-acpr.hessianpzy/(acpr.iomega*rho_w))
+conj(acpr.vz)*(-acpr.hessianpzz/(acpr.iomega*rho_w))

To utilize the calculated PRF and VRF in the 'particle tracing' module adopted for specific particles in a taSSAW configuration, they should be transferred to local CS through the following expressions in which $tetaR$ is the inclination angle of the acoustofluidic device.

PRF_xt = cos(tetaR)*PRF_x+sin(tetaR)*PRF_y
PRF_yt = cos(tetaR)*PRF_y-sin(tetaR)*PRF_x (S18)
PRF_zt = PRF_z

VRF_xt = cos(tetaR)*VRF_x+sin(tetaR)*VRF_y
VRF_yt = cos(tetaR)*VRF_y-sin(tetaR)*VRF_x (S19)
VRF_zt = VRF_z

Appendix C. Additional Information, Figures and Movies for the Main Text

Mesh element type and size

The maximum permissible mesh element size in the simulation of the acoustic and streaming field with free-triangular mesh type equals to $\lambda/6$ based on a mesh dependency study presented by Lei et al. (6) in which varying mesh size from $\lambda/6$ to $\lambda/20$ would lead to a maximum 5% error reduction. In order to balance the numerical accuracy and computational load, the mesh element size of $\lambda/6$ is adopted throughout of the presented study which conforms with the COMSOL documentation and other analogous investigation in the literature as well (4, 7). Utilizing the periodic boundary condition requires completely identical mesh type and distribution on the left and right boundaries (Γ_L, Γ_R) of the computational domain (Fig. 1b in the main text). Therefore, with utilizing sweep technique and arithmetic pattern, the prismatic elements are developed to the substrate depth (Fig. S3).

For the harmonic analysis, the free-triangular surface mesh with the maximum permissible element size is developed to the depth of the substrate with free-tetrahedral mesh elements (Fig. S3); since the convergence rate and accuracy of free-tetrahedral mesh elements are higher compared to prismatic ones generated in the sweep technique. Our simulation results demonstrate that in a similar case, utilizing free-tetrahedral elements despite increasing degrees of freedom could approximately reduce the computational time by a factor of one-sixth. Moreover, the free-tetrahedral mesh elements with maximum permissible mesh element size ($\lambda/6$) are utilized for the fluid cavity due to their higher convergence rate.

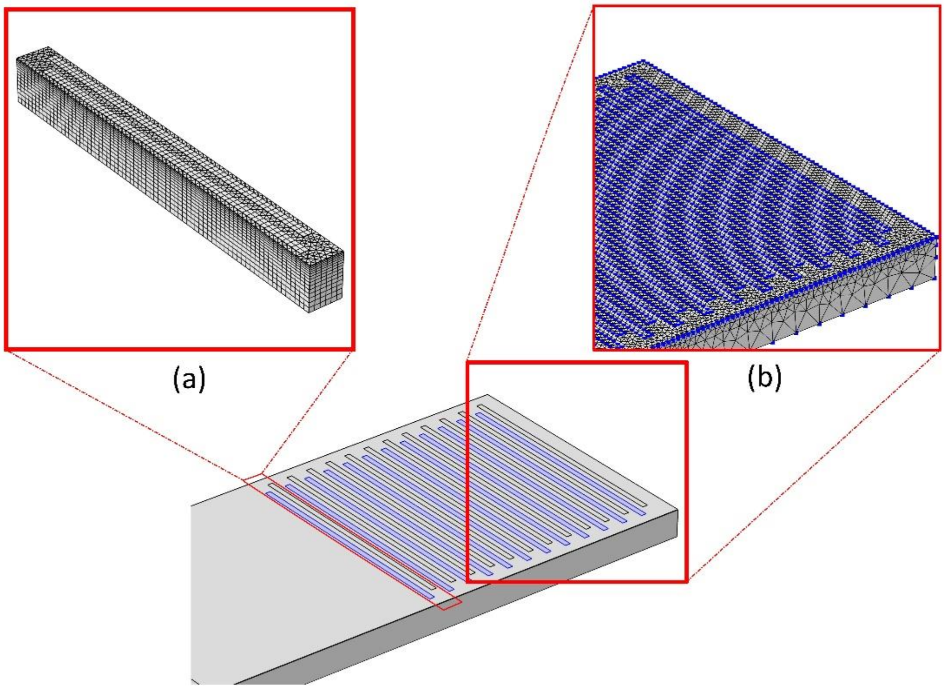

Figure S 3. (a) The meshed structure of reduced domain with prismatic mesh elements, (b) The meshed structure of full-domain with free-tetrahedral mesh elements

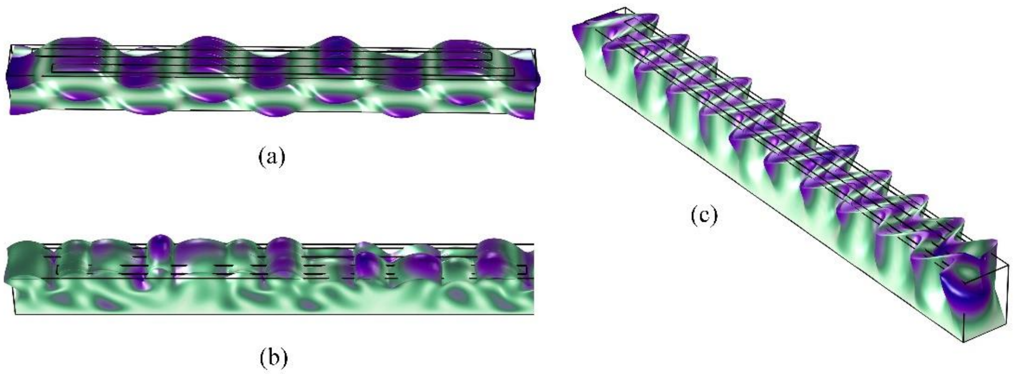

Figure S 4. Sample vibration mode shapes that no SAW of Rayleigh type would be generated in them due to the particle polarization direction (a,c) and penetration of particle polarization in substrate depth (a,b)

Boundary condition

This section is devoted to the explanation of utilized boundary conditions for solving Helmholtz equation (Eq. 8 in the main text) and capturing fluid-solid interaction. The dimensions of PDMS microchannel are generally considered larger than the fluid cavity to reduce the influence of the wave reflection from air/PDMS interface to the fluid domain for which a threshold of 2 mm thickness or greater is reported in the literature (8). Consequently, to reduce the computational cost, the channel walls are not included in the presented simulation, while their impression on the induced acoustic field in the microchannel is considered through implementing proper boundary conditions. As shown in Fig. 1c in the main text, in line with Ding et al. (9), a PDMS microchannel with a rectangular cross-section of w=1000 µm and h=75 µm is considered here, where the boundaries in contact with the PDMS wall are denoted with Γ_L, Γ_R, Γ_T, and their influence on the acoustic field is modeled with impedance or lossy-wall boundary condition. The impedance boundary condition can be formulated as a planar wave propagates from water to the surrounding medium of density ρ_m and speed of sound c_m expressed as follows (10),

$$n_i p_{1,i} = -i \frac{\omega \rho_f}{\rho_m c_m} p_1 \qquad \text{(S20)}$$

where n_i is the interface normal vector pointing to the adjacent medium. The utilization of impedance boundary conditions is only valid under the assumption that the thickness of the PDMS channel wall should be large enough to attenuate a significant amount of transmitted wave energy. For commonly-used PDMS (10: 1) at room temperature, the spatial attenuation coefficient (α_s) would be 47.85 dB/cm and 64.33 dB/cm for 9 MHz and 11 MHz frequencies, respectively, leading to the 1102 NP/m and 1482 NP/m decay coefficients (11). Therefore, for the considered 9.63 MHz operating frequency, 3 mm thickness of the PDMS channel wall leads to the approximately 99% absorption of acoustic waves in the channel wall for which, as shown in Fig. 1a in the main text, enough space is anticipated in the microdevice designing step.

Once the simulated medium interfaces a neighboring liquid cannot tolerate any applied pressure, like a free liquid/air interface; the soft-wall boundary condition is utilized (10). This boundary condition is applied on the inlets and outlet of the microchannel in the presented simulation for solving the Helmholtz equation (Eq. (8) in the main text), and mathematically is stated as,

$$p_1 = 0 \qquad \text{(S21)}$$

Another vital aspect of acoustofluidic processes' simulation is the acoustic coupling between interacting fluid and solid components of the device implemented by imposing continuity condition on velocity (Eq. (S22)) and traction vector (Eq. (S23)) in the interface normal direction depicted by Γ_B in Fig. 1c of the main text. This boundary condition can be applied through a predefined node in COMSOL Multiphysics 5.3a known as 'acoustic-structure boundary' based on the following equations for inviscid fluid-structure interaction (12).

$$i\omega u_i n_i = v_{1i} n_i \tag{S22}$$

$$T_{ij} n_j = -p_1 n_j \tag{S23}$$

Before conducting the particle separation in acoustofluidic devices with an established SSAW field, it is required to focus and align microparticles, in line with Ding et al. (9), done hydrodynamically with side sheath flows in the presented simulation. Thus, two side inlets (Fig. 1a and Fig. 1c of the main text) with 200 µm and 500 µm width and flow velocity of 12 mm/s and 24 mm/s, respectively, are considered for the focusing of particles. Based on the conducted simulation, with the aforementioned velocities for sheath flows, the induced downstream flow would reach ~15 mm/s velocity before entering the SSAW separation region, approximately coinciding with Ding et al. (9) investigation. To ensure that Rayleigh SSAW would propagate in the piezoelectric substrate (YX 128° $LiNbO_3$), it is vital to perform a frequency analysis (modal and harmonic analysis) around 19.4 MHz operating frequency utilized by Ding et al. (9). Following the procedure explained in the main text leads 19.32 MHz operating frequency.

Computational Cost

To demonstrate the cost and time efficiency of the presented 3D fully-coupled model, the best means is to make a comparison between available 3D models of SAW microfluidics according to the required computational time and resources with regarding the complexity of examined on-chip configuration.

The first model is proposed by Skov et al. (13) in which they employed the traditional perturbation approach, and solved second-order equations in a fluid medium encompassing IDTs. Due to the considering viscous boundary layer, their 3D model contains 4.6 million degrees of freedom. The calculation is distributed across 80 nodes on the HPC and takes 14 hours to compute; even though, they utilized PML and antisymmetric technique to scale down the model. Their computational domain was a $0.5 \times 0.9 \times 1.2$ mm cubic body of water (with 0.05 mm height) and Pyrex/PDMS channel wall on a piezoelectric substrate of $0.3 \times 1.4 \times 1.2$ mm.

The second model is developed by Chen et al. (7) in which they solve second-order equations in a layer by thickness of $4\delta_v$ (δ_v is viscose boundary layer thickness) adjacent to IDTs, and utilized the result of preceding step to calculate slip velocity just outside of the first layer. This slip velocity would serve as boundary condition to solve creeping flow in bulk of the fluid. They did not provide any information about computational load; however, in the appendix, they state that this method on a 2D model, which is presented as verification of the method, can save time up to 53% compared to the traditional perturbation approach. On a similar simulation condition with last discussed model, their model approximately would need 80 nodes on a HPC for 7 hours.

In the presented study, we consider the verification model, as the most demanding model, which is approximately a cubic computational domain of water with $1 \times 0.07 \times 12$ mm on a

piezoelectric substrate of $13 \times 19.6 \times 0.5$ mm. The volume of our computational domain and substrate are approximately 1.5 times and 252.7 times larger than the first mentioned model, respectively. Note that in the discussed simulations, no water flow is considered, and the configuration of the channel and IDTs are relatively simpler. The run of the mentioned model took about 5 hours on 12 cores. The computational time can decrease by increasing the number of utilized cores on the processor; however, the required queue time is elevated proportionally. Therefore, by utilizing 80 cores on HPC, the mentioned model's computation would take approximately less than 1 hour. Thus, roughly speaking, to get a sense about the efficiency of the presented model, on a relatively more complex and larger on-chip configuration, the presented fully-coupled 3D model can save time up to 93%.

To be more accurate, the computing was performed on high-performance cluster computing (HPCC) facilities running on Linux operating system equipped with AMD Opteron 6174 processor (2.2 GHz clock frequency) along with a graphic processor unit (GPU) for highly demanding models. Different models are analyzed during the presented study; therefore, for the sake of brevity, the required computational time and resources only for the most expensive model (verification model) are explained here. The first and most expensive step is to calculate the first-order acoustic field in the whole computational domain (Fig. 1a in the main text), which leads to about 6.5 × 106 degrees of freedom, a calculation time of 212 min, and a peak RAM usage of 68.5 GB or 71% with the utilization of 12 cores on HPCC. The second step calculation, solving creeping flow with limiting velocity as a slip boundary condition in a fluid medium (Fig. 1c in the main text), required around 1.4 × 106 degrees of freedom and took 46 min while having a peak RAM usage of 17.3 GB or 18%. The third step is to calculate the total 400 particle trajectories for polystyrene beads of 9.9 µm and 7.3 µm diameter, which takes 53 min and a peak RAM usage of 13.3 GB.

Equivalent input power calculation

In piezoelectric materials, the electric polarization density ($\boldsymbol{P} = (P_i)$) according to the external strain is linear ($P_i = e_{\boldsymbol{ikl}} S_{kl}$). Thus, from inverse piezoelectric effect constitutive relation, we have,

$$D_i = \varepsilon_{ij} E_j + P_i \tag{S24}$$

When the polarization density changes with time, the time-dependent bound-charge density creates a polarization current density ($\boldsymbol{J_p} = \partial \boldsymbol{P} / \partial t$). By calculating the integration of current density over the surface piezoelectric substrate in the electrodes' place, it is feasible to derive an equivalent current (I_{eq}).

$$I_{eq} = \iint_A \boldsymbol{J_P} . d\boldsymbol{A} \tag{S25}$$

By utilizing the notion of root mean square AC voltage ($V_{rms} = V_0/\sqrt{2}$) and calculated equivalent current, the total equivalent applied input power (W_t) would be calculated as follows,

$$W_t = W_{el+} + W_{el-} = \sqrt{2} V_0 I_{eq} \quad \text{(S26)}$$

The aforementioned calculations are based on the premise that the internal losses of piezoelectric material are neglected in the presented simulation.

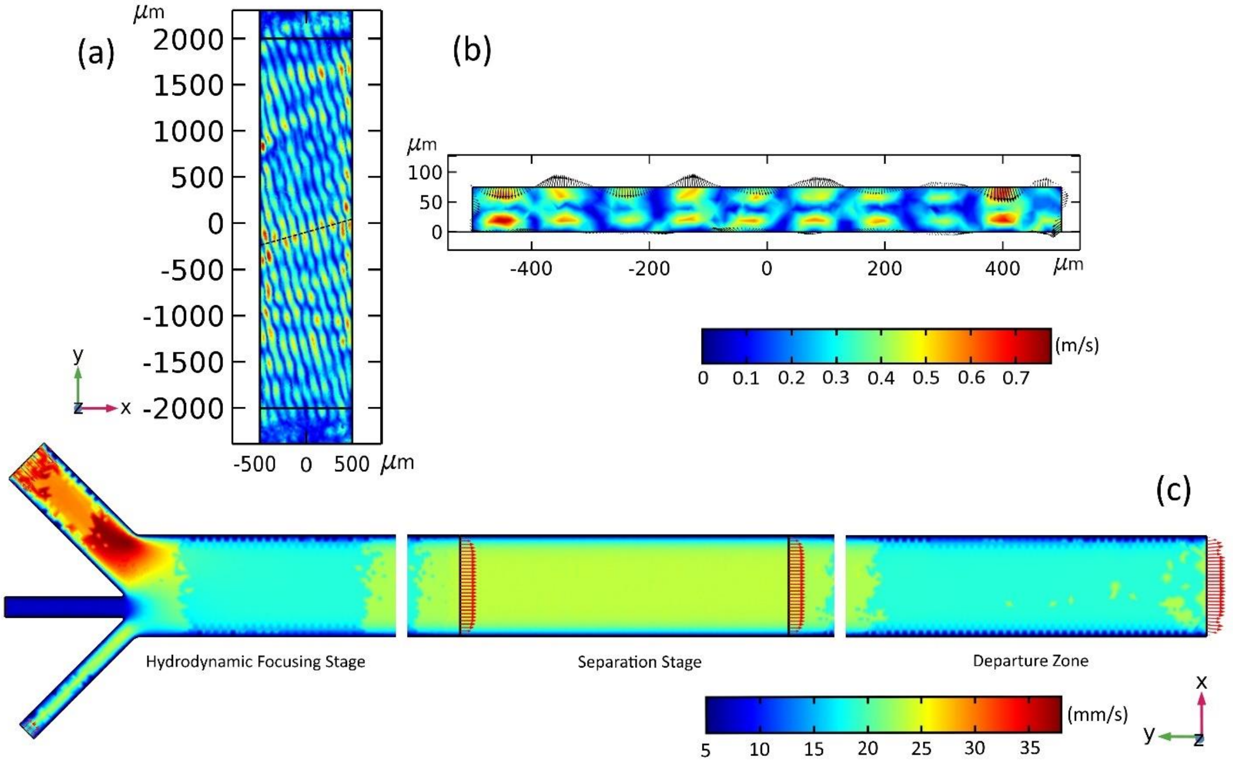

Figure S 5. (a) First-order velocity field in the xy plane with 20 μm height from the bottom of the channel along the z-axis, (b) first-order velocity field in the xz cross-section of the channel along the depicted dashed line in a, (c) second-order velocity field in the xy plane on the channel and substrate interface.

Regarding Fig. S5, the first-order and second-order velocity fields for the verification model under 7.5 V driving voltage (26.5 dBm input power) are depicted. The first-order velocity field ($\boldsymbol{v_1}$) is calculated from the first-order acoustic pressure field (p_1) based on Eq. (9) in the main text, and, consequently, as shown in Fig. S5a, it preserves the tilted pattern of the first-order acoustic pressure field in Fig. 5b of the main text. The velocity vector and contour of the first-order velocity field along the depicted dashed line in Fig. S5a, perpendicular to the pressure nodal lines, are presented in Fig. S5b in the xz cross-section of the channel. Note that the indicated magnitude scale-difference between time-harmonic and time-averaged quantities is observed between first-order and second-order velocities as well. The second-order velocity field ($\boldsymbol{v_2}$) on the channel and substrate interface is depicted in Fig. S5c. Due to the higher magnitude of limiting

velocities in the separation zone (working zone), the second-order velocity magnitude is higher in this section than adjacent ones. The propagated SSAW leaked from the working zone to the adjacent zones (focusing and departure stage in Fig. S5c); therefore, the limiting velocity is calculated in the adjacent zones, and its influence on the fluid flow is captured in the presented simulation. The fluid flow in the presented simulation is affected by outer acoustic streaming and syringe-pump-driven streaming, both of which are captured by adopting appropriate boundary conditions, including limiting velocity method and constant inlet flow rate. However, due to the applied high inlet flow velocity according to the simulated experimental setup, syringe-pump-driven streaming dominates the net fluid flow pattern in the presented simulation. The inlet and outlet disturbance are observed in the second-order velocity field (Fig. S5c).

Sheathless particle separator utilizing taSSAW advantages and justification

Utilization of the presented on-chip configuration can alleviate the following shortcomings of the traditional hydrodynamic focusing approach. First of all, it would diminish the diluting impression of the sheath flows on the analytes by lowering the required flow rate of the introduced side flow due to its attributing function, which is limited to stream conducting instead of the whole particle focusing, and aligning process in precedent platforms. Moreover, a relatively complicated design procedure is required in traditional platforms to generate sheath flows with satisfactory conditions and, subsequently, enhance separation efficiency, which should be conducted for each sheath liquid composition on a case-specific basis. In hydrodynamic focusing, the precise flow control between sample and sheath flows is an indispensable part of the process that should be handled by parallel or separate high-precision syringe pumps to ensure consistent pressure and flow rate. However, in the presented configuration, the function of the introduced side flow is restrained to stream realignment for which the precedent accuracy and sensitivity would not be required which in turn could lead to increased reliability of the device, and with good design, the whole process can be managed with one syringe pump.

Furthermore, in conventional hydrodynamic focusing, increasing the flow rate higher than a few hundred microliters per minute would lead to decreasing the overall resolution of the device; however, implementing acoustic-assist focusing allows high resolution at a high flow rate, which could speed up the whole on-chip process (14). The proposed configuration also would have the capability of running multiple parallel flow streams with a multinode acoustic focusing technique (14), which enables the possibility of analyzing more than two cells/particles in one separation cycle.

Regarding the complexity of running the set-up, in the designing step of sheathless particle separator, all the characteristics of induced SAW on the concentration and separation stage are considered identical (driving frequency = 9.63 MHz, phase difference = π, waveform = sine wave) except wave amplitude which contributes to the input power or driving voltage. Most signal generators are of the two output channels which can be utilized to feed two IDTs, especially when

the low input power required for actuation of SAWs, which is reaching to maximum 33 dBm = 2 W here, and the main characteristics of them are identical. Therefore, no extra accessories like signal generator or amplifier are required to run the device, and one two-channel signal generator would suffice. In summary, the proposed configuration would have advantages, including diminishing diluting impression, removing high-precision syringe pump, more straight forward designing procedure, more reliability of the device, increased concentration resolution in high flow rate, multinode acoustic focusing capability, and more miniaturing feasibility. Therefore, owing to achieving aforementioned improvement without any extra notable accessories compared to conventional hydrodynamic set-up, the proposed sheathless separator is justifiable.

Inclination angle (θ) optimization

As the first designing step of the taSSAW based sheathless platform for the separation of 4 µm and 10 µm PS particles, the inclination angle (θ in Fig. S6 and Fig. 1d of the main text) should be optimized for different driving voltages (input powers) to achieve the maximum lateral inter-particle distance after egressing the separation stage which in turn would improve the separation efficiency of the device. It is beneficial to exclude focusing and realignment stages in the optimization process to alleviate the computational cost and assumed that the focused aligned suspended particles would enter the working region of taSSAW for the separation purpose with an initial flow velocity of 10 mm/s (Fig. S6). Under this condition, a fraction of the ultrasonic wave energy would leak through radiation into the omitted adjacent parts of the fluid domain. Based on the explained concept of the acoustic impedance, planar acoustic wave radiation into a neighboring medium that occurred in the inlet and outlet of the considered channel section could be modeled through impedance boundary condition (Eq. S20) to solve the Helmholtz equation (Eq. 8 in the main text). Other required boundary conditions and utilized meshing technique would be analogous to presented model verification.

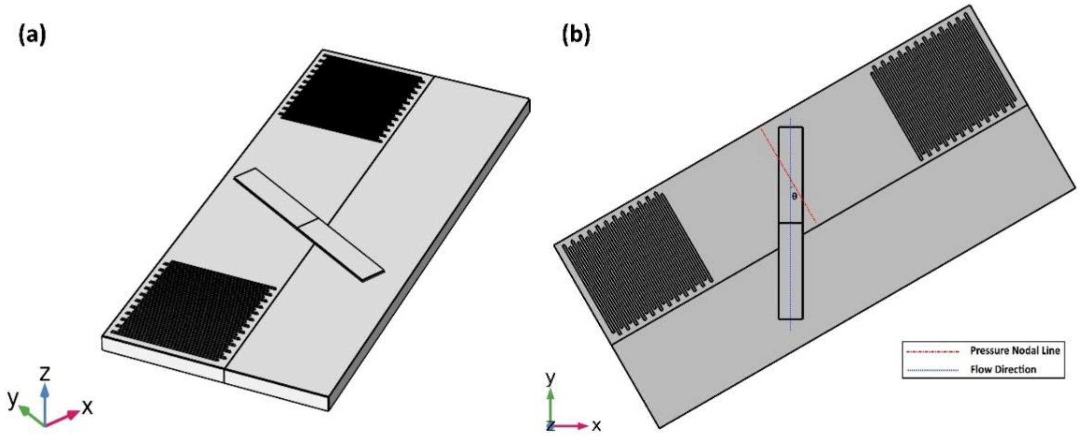

Figure S 6. (a) Isometric view of the 3D model in inclination angle optimization for θ=30°, (b) xy cross-section of the model in which the inclination angle (θ) is depicted.

In the presented optimization process, the inclination angle (θ) is variated from 5° to 45° for three different driving voltages (input powers), including 20 V (26.5 dBm), 30 V (30 dBm), and 40 V (33 dBm). In each one, the maximum separation inter-particle distance after egressing the working region is determined, for which the captured results are presented in Fig. 7 of the main text.

As shown in Fig. 7 of the main text, the general trend of the conducted optimization results is that increasing the inclination angle (θ) between the pressure nodal lines and flow direction would decrease maximum lateral separation distance at the channel outlet in all the considered power levels. While increasing the driving voltage would provoke a more intense acoustic radiation force, which breeds higher separation distance under similar conditions. Nevertheless, for each input power, there is an abrupt increase in lateral separation distance after θ reaches a particular value, which is 10°, 20°, and 40° for 40 V (33 dBm), 30 V (30 dBm), and 20 V (26.5 dBm) driving voltage, respectively. Note that by decreasing the input power level, contributing abrupt variation, which is of utmost importance in improving the device's separation efficiency, would occur in higher inclination angles. Therefore, for low input powers, the indicated abrupt increase conceivably could not dominate the general decrement influence of higher inclination angles on separation distance, such as what is observed for 20 V driving voltage in Fig. 7 of the main text, which demonstrates the maximum separation distance with θ = 5° ($\sim$ 250 µm) is more than that of with θ = 40° ($\sim$ 150 µm) corresponding to the abrupt increment.

The variation of the particles' lateral displacement with time along the width of the microchannel for 20 V, 30 V, and 40 V driving voltage and under 5°, 20°, and 10° inclination angle, respectively, for which the maximum separation distance would be attained based on the optimization results, is presented in Fig. S7. The simulated trajectory of PS beads with 4 µm diameter have shown negligible dependency on driving voltage; thus, it is depicted regardless of driving voltage and input power in the following plot. The maximum inter-particle separation distance in each applied driving voltage at the channel outlet is denoted with δ. As observed in Fig. S7 and indicated before, the particles' applied input power and maximum separation distance have a direct relationship. Based on the optimization results, 40 V (33 dBm) driving voltage under 10° inclination angle is utilized to separate PS beads of 4 µm and 10 µm diameter in the simulation of the proposed sheathless configuration, and presented geometrical dimensions in Fig. 6b of the main text is based on this optimum state. For the sake of brevity, simulated particle trajectories, pressure nodal lines, limiting velocity, and second-order quantities are just presented for the indicated optimum state in Fig. S8.

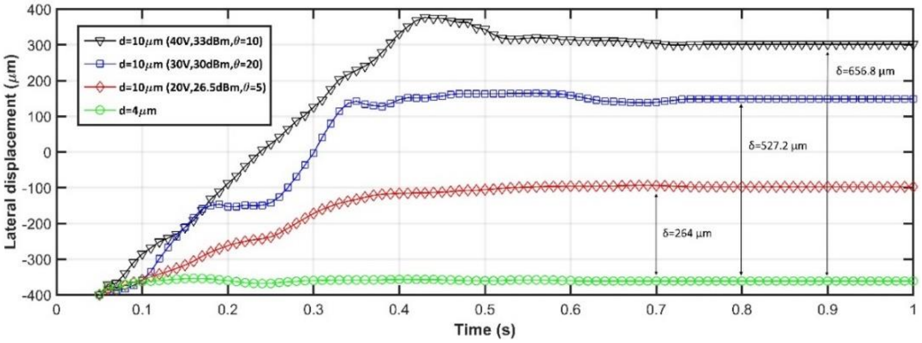

Figure S 7. Lateral displacement of PS beads with 10 μm and 4 μm diameter vs. time along the width of the channel in 20 V, 30 V, and 40 V driving voltage and under 5°, 20°, and 10° inclination angle, respectively.

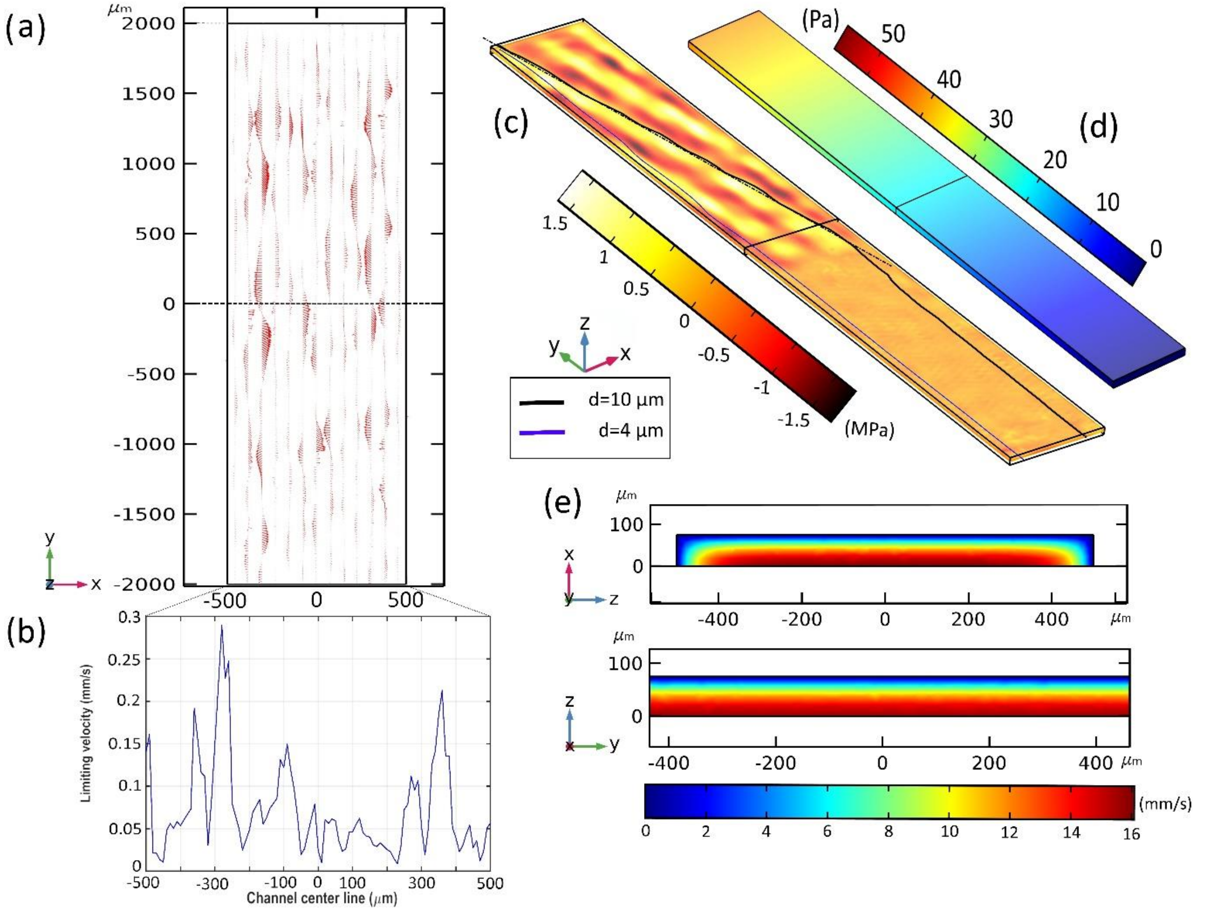

Figure S 8. The simulation results of 40 V driving voltage and θ= 10° (optimum state), (a) the calculated limiting velocity field on the channel interface in the taSSAW working region, (b) the magnitude of limiting velocity along the horizontal center line of the channel depicted with dashed line in a, (c) The isometric 3D view of microchannel, acoustic pressure nodal lines, and particle trajectories in the taSSAW field, (d) second-order pressure field of the microchannel, (e) second-order velocity field in the bulk of the fluid for xz and yz cross-sections.

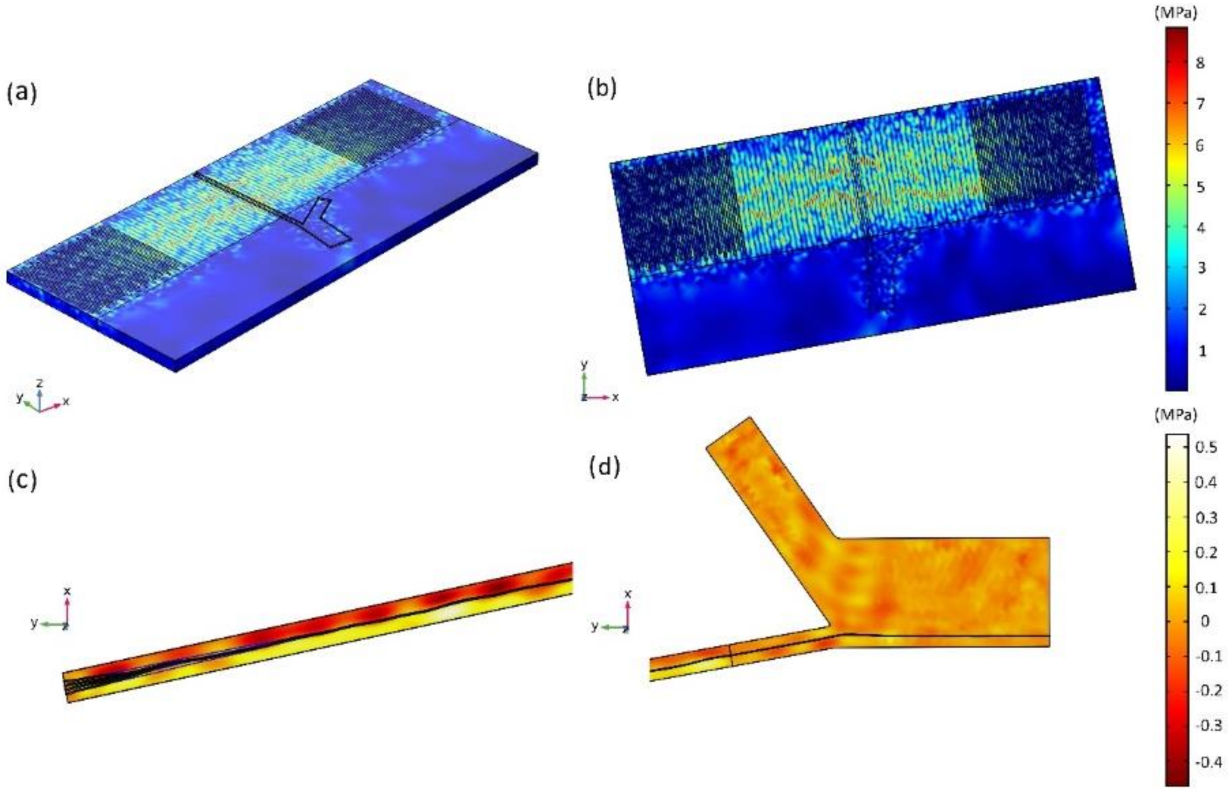

Figure S 9. (a) The isometric 3D view of the full model and the pressure lines of the stress field in the LiNbO3 substrate, (b) xy cross-section of the utilized full model, (c) the focusing of PS particles along the first-order pressure nodal line in the fluid domain, (d) the realignment of focused PS particles with side flow before entering separation stage

Regarding Fig. S9, note that the maximum stress value in the substrate reaches 8 MPa; however, after transferring to the fluid domain, the acoustic pressure field's absolute peak value reaches 0.5 MPa. As depicted in Fig. S9c and Fig. S9d, the concentration of suspended particles along the pressure nodal line in the focusing stage and the suspended particles' realignment are well-captured in the conducted simulation.

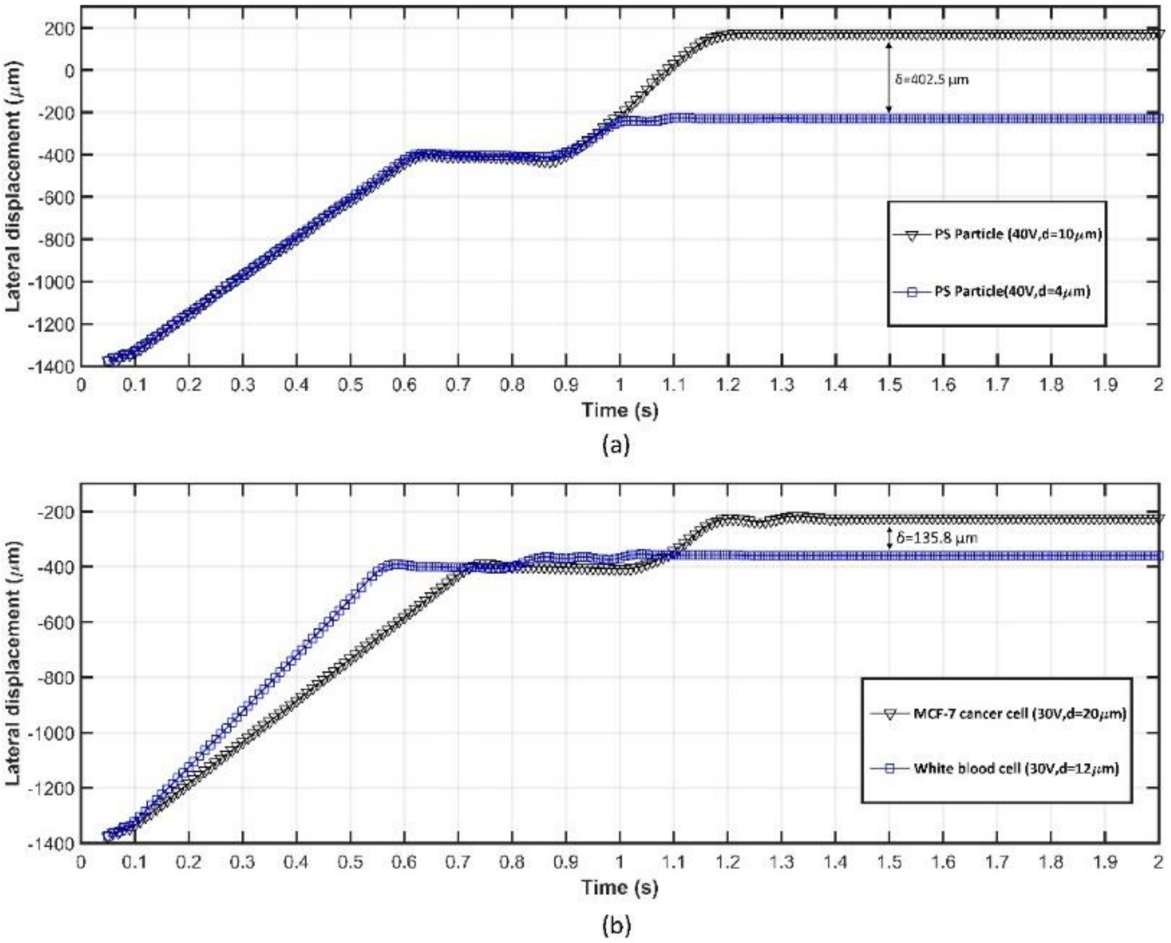

Figure S 10. The lateral displacement during the simulation of sheathless platform for, (a) the PS particles (40 V, ϑ= 10°), and (b) the WBCs and MCF-7 cancer cells (30 V, ϑ= 10°)

Regarding Fig. S10, the coincidence of PS particles' trajectories in the focusing stage (until ~ 0.6 s in Fig. S10a) and realignment stage (from ~ 0.6 s to ~ 0.9 s in Fig. S10a) is noticeable. On the contrary, it takes longer for MCF-7 cancer cells to end up focusing stage (about 0.7 s in Fig. S10b) relative to WBCs (about 0.55 s in Fig. S10b), which arises since the Stokes drag force (DF) is proportional to the cell/particle diameter (Eq. 14 in the main text), and more effective DF on cancer cells leads to the decrement of their flow velocity.

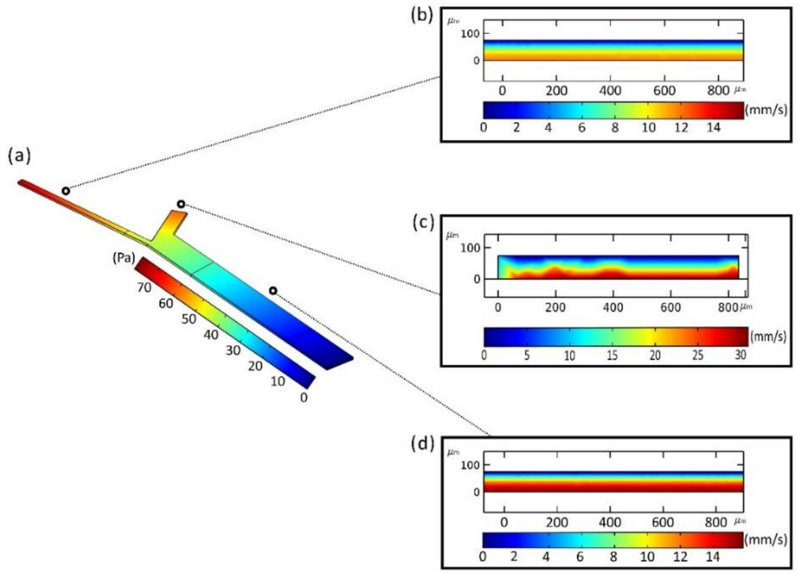

Figure S 11. In the PS particles' simulation, (a) The 3d view of the second-order pressure distribution, the second-order velocity profile in the (b) focusing stage, (c) realignment stage, and (d) separation stage

Regarding Fig. S11, the second-order time-averaged pressure and velocity field in the bulk of the fluid domain is presented just for PS particles in Fig. S11. As shown in Fig. S11a, the highest value of second-order pressure reaches 70 Pa in the focusing stage, while, as stated before, the first-order pressure can rise to the maximum value of 0.5 MPa (Fig. 8a of the main text), which conforms with the indicated scale-difference between the magnitude of time-harmonic and time-averaged quantities. The second-order velocity profile of the focusing stage is presented in Fig. S11b with considering 800 µm of microchannel length and a perpendicular cross-section on the fluid-substrate interface. The utilized cross-sections for demonstrating the second-order velocity profile in the realignment stage (Fig. S11c) and separation stage (Fig. S11d) are defined with the analogous procedure. The maximum value of the second-order velocity in each stage occurs in the vicinity of the fluid-substrate interface because of applying slip boundary conditions on it, namely 11 mm/s for the focusing stage, 30 mm/s for the realignment stage, and 14 mm/s for the separation stage. The observed initial disturbance in the second-order velocity profile of the realignment stage relative to the fully developed one in the focusing and separation stage can be attributed to the fact that the lower induced limiting velocity field on the realignment region interface could not dominate over the more intense initial disturbance provoked by applying higher inlet velocity. The lower limiting velocity arises since the realignment stage is located out of the two established standing acoustic fields (Fig. 6 of the main text).

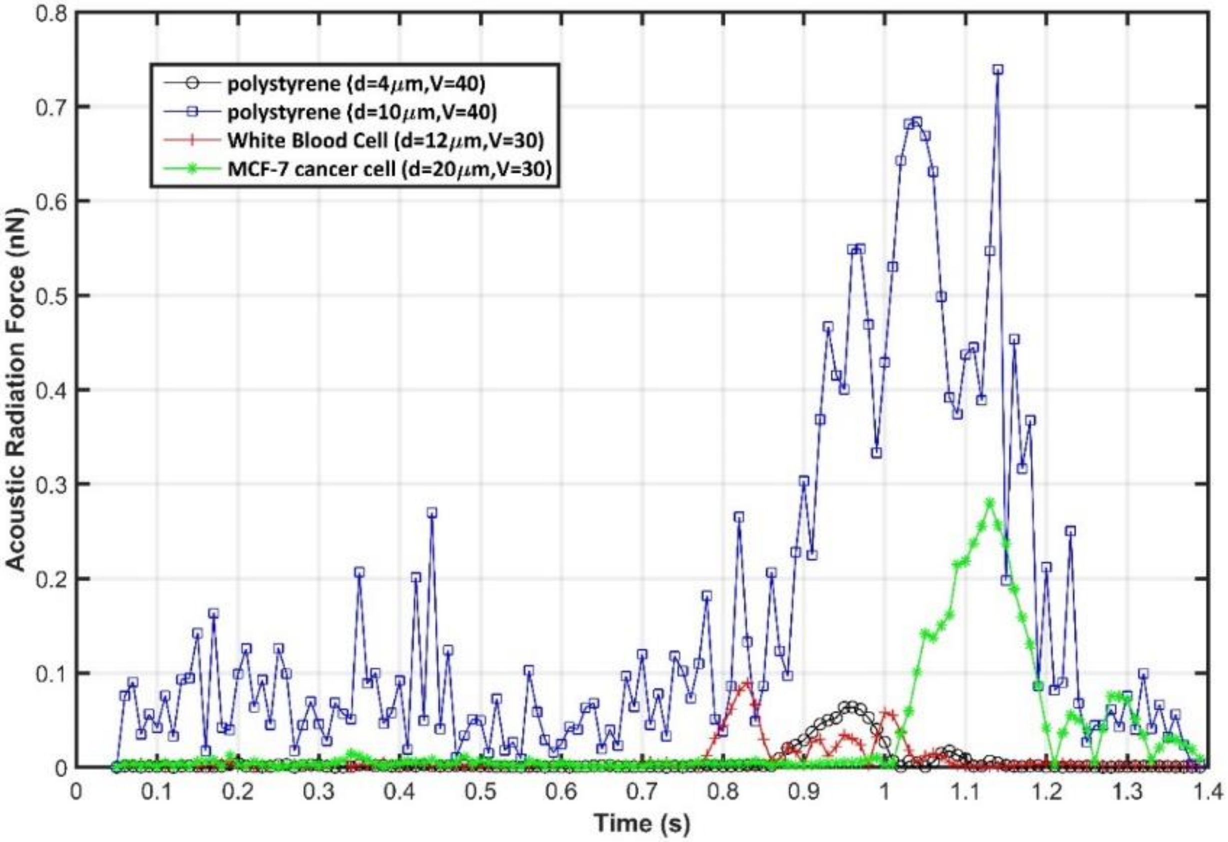

Figure S 12. The magnitude of applied ARF on the PS particles of 4 μm and 10 μm diameter under 40 V driving voltage, and WBCs and MCF-7 cancer cells with 12 μm and 20 μm diameter under 30 V driving voltage

Regarding Fig. S12, the maximum ARF is applied on the PS particles with 10 μm diameter, and under 40 V driving voltage, after that, the cancer cells, WBCs, and PS particles with 4 μm dimeter would experience more intense ARF in descending order. Therefore, despite the proportionality of ARF on the target cell/particle's volume, more diameter would not necessarily lead to more ARF, and other parameters like input power level (driving voltage) and particle's properties (density and compressibility) are effective on the magnitude of ARF which are captured in the presented simulation based on Eq. 12 and Eq. 13 in the main text.

Movie S1. Simulation results of separating polystyrene (PS) beads (7.3 µm and 9.9 µm diameter) with taSSAW field, and hydrodynamic focusing approach under 15° inclination angle, 19.32 MHz operational frequency, and 26.5 dBm input power. This movie corresponds to Fig. 4 of the main text.

Movie S2. Simulation results of separating PS beads (4 µm and 10 µm diameter) with taSSAW field, and SSAW focusing approach in the proposed sheathless platform under 10° inclination angle, 9.63 MHz operational frequency, and 33 dBm input power. This movie corresponds to Fig. 8a and Fig. 8b of the main text.

Movie S3. Simulation results of separating WBCs (12 µm diameter) from MCF-7 breast cancer cells (20 µm diameter) with taSSAW field, and SSAW focusing approach in the proposed sheathless platform under 10° inclination angle, 9.63 MHz operational frequency, and 24.7 dBm input power. This movie corresponds to Fig. 8c and Fig. 8d of the main text.

References

1. Gantner A, Hoppe RH, Köster D, Siebert K, Wixforth A. Numerical simulation of piezoelectrically agitated surface acoustic waves on microfluidic biochips. Computing and Visualization in Science. 2007;10(3):145-61.
2. Ledbetter H, Ogi H, Nakamura N. Elastic, anelastic, piezoelectric coefficients of monocrystal lithium niobate. Mechanics of materials. 2004;36(10):941-7.
3. Auld BA. Acoustic fields and waves in solids: Рипол Классик; 1973.
4. COMSOL A. COMSOL Multiphysics 5.3 User's Guide. COSMOL, Inc. Burlington, MA; 2006.
5. Hahn P. Numerical simulation tools for the design and the analysis of acoustofluidic devices: ETH Zurich; 2015.
6. Lei J, Glynne-Jones P, Hill M. Acoustic streaming in the transducer plane in ultrasonic particle manipulation devices. Lab on a Chip. 2013;13(11):2133-43.
7. Chen C, Zhang SP, Mao Z, Nama N, Gu Y, Huang P-H, et al. Three-dimensional numerical simulation and experimental investigation of boundary-driven streaming in surface acoustic wave microfluidics. Lab on a Chip. 2018;18(23):3645-54.
8. Nama N, Barnkob R, Mao Z, Kähler CJ, Costanzo F, Huang TJ. Numerical study of acoustophoretic motion of particles in a PDMS microchannel driven by surface acoustic waves. Lab on a Chip. 2015;15(12):2700-9.
9. Ding X, Peng Z, Lin S-CS, Geri M, Li S, Li P, et al. Cell separation using tilted-angle standing surface acoustic waves. Proceedings of the National Academy of Sciences. 2014;111(36):12992-7.
10. Bruus H. Acoustofluidics 2: Perturbation theory and ultrasound resonance modes. Lab on a Chip. 2012;12(1):20-8.
11. Tsou JK, Liu J, Barakat AI, Insana MF. Role of ultrasonic shear rate estimation errors in assessing inflammatory response and vascular risk. Ultrasound in medicine & biology. 2008;34(6):963-72.
12. Nam J, Lim H, Kim C, Yoon Kang J, Shin S. Density-dependent separation of encapsulated cells in a microfluidic channel by using a standing surface acoustic wave. Biomicrofluidics. 2012;6(2):024120.

13. Skov NR, Sehgal P, Kirby BJ, Bruus H. Three-Dimensional Numerical Modeling of Surface-Acoustic-Wave Devices: Acoustophoresis of Micro-and Nanoparticles Including Streaming. Physical Review Applied. 2019;12(4):044028.
14. Piyasena ME, Austin Suthanthiraraj PP, Applegate Jr RW, Goumas AM, Woods TA, López GP, et al. Multinode acoustic focusing for parallel flow cytometry. Analytical chemistry. 2012;84(4):1831-9.
